# Lettuce immune responses and apoplastic metabolite profile contribute to reduced internal leaf colonization by human bacterial pathogens

**DOI:** 10.1186/s12870-025-06636-1

**Published:** 2025-05-14

**Authors:** Cristián Jacob, Maeli Melotto

**Affiliations:** 1 Departamento de Ciencias Vegetales, Facultad de Agronomía y Sistemas Naturales, Pontificia Universidad Católica de Chile, Santiago, Chile; 2 Department of Plant Sciences, University of California, Davis, CA USA

**Keywords:** Food safety, Fresh produce, Plant-microbe interaction, *Salmonella enterica*, *Escherichia coli*, Leaf Apoplast, Plant defense, Omics

## Abstract

**Background:**

Human bacterial pathogens such as *Salmonella enterica* and *Escherichia coli* can colonize the apoplast of leafy greens, where they may evade standard sanitization measures and persist until produce consumption. Bacterial survival in this niche is influenced by plant immune responses that may vary according to bacterial species and plant genotypes. The variability in immune responses has been associated with differences in pathogen persistence capacity within the phyllosphere. In addition, emerging evidence suggests that preexisting and inducible plant metabolites contribute to either restricting or facilitating colonization of human pathogens in plant tissues. Identifying the molecular mechanisms underlying these interactions is crucial for developing strategies to mitigate contamination in fresh produce.

**Results:**

We characterized whole-leaf transcriptome and apoplast metabolome profiles of three lettuce cultivars upon exposure to the human pathogenic bacteria *S. enterica* ser. Typhimurium 14028s and *E. coli* O157:H7. The lettuce genotypes Lollo Rossa and Green Towers exhibited stronger transcriptional modulation, primarily associated with defense-related processes and showed reduced bacterial survival in their apoplast. Surprisingly, Green Towers did not generate callose deposition or reactive oxygen species burst responses at levels comparable to that of Lollo Rossa, suggesting it has distinct modifications in the apoplastic conditions that restrict pathogen persistence. Apoplastic metabolomic profiling revealed specific compounds alterations in Green Towers linked to bacterial survival, indicating their potential role in this genotype’s defense mechanism. In contrast, the lettuce cultivar Red Tide exhibited minimal transcriptional and metabolic modulation, lack of robust defense activation, which was accompanied by apoplastic bacterial survival.

**Conclusions:**

This study provides evidence that lettuce cultivars exhibit distinct molecular responses that may influence the persistence of human bacterial pathogens in the leaf apoplast. The results indicate that both immune response activation and metabolite composition may contribute to restrict apoplastic bacterial persistence or growth. These findings offer novel insights into the genetic and biochemical factors shaping lettuce-pathogen interactions, which might inform breeding programs and agronomic practices aimed at enhancing food safety.

**Supplementary Information:**

The online version contains supplementary material available at 10.1186/s12870-025-06636-1.

## Introduction

The apoplast encompasses all compartments beyond the plasmalemma, including the cell wall, intercellular spaces, and the xylem [1]. This dynamic environment contains gas, liquid, and a diverse range of metabolites [2, 3] and plays a central role in essential processes such as gas exchange between mesophyll cells and the atmosphere [2], nutrient uptake and solute transport [4], cell wall assembly [5], external signal perception [6], and intercellular communication [7]. The apoplast is also a key site for plant-microbe interactions [8], where pathogens and endophytic microbes can colonize foliar tissues, forming pathogenic, commensal, or mutualistic associations with the host plant [9]. Phyllosphere microbial communities can contribute to plant health [10, 11] and might influence human health through potential links with the human gut microbiome [12]. However, foliar tissues may also serve as reservoirs for human pathogens, which might result in foodborne illness outbreaks through consumption of contaminated fresh produce [13, 14].

To thrive in the apoplast, microbes must suppress or evade plant immune responses [9, 15], acquire nutrients, and manipulate host cell physiology to promote the release of water and nutrients [16, 17]. In response, plants activate defense mechanisms, including microbe-associated molecular patterns (MAMPs) perception, reactive oxygen species (ROS) bursts, stress hormone signaling, pathogenesis-related protein secretion, and secondary metabolite production [18]. These are fast defense responses that alter the biochemistry and physiology of the apoplast early in plant-human pathogen interactions. For example, lettuce exposed to *Salmonella enterica* serovar Typhimurium 14028s or *Escherichia coli* O157:H7 shows ROS burst and callose deposition [19, 20]. Interestingly, the extent of defense responses varies across different human bacteria and plant cultivars, at both inter- and intra-specific levels [19, 21–23] and this variability is associated with human pathogen population sizes in the phyllosphere [19].

Bacteria possess remarkable metabolic adaptation; however, the composition and availability of environmental nutrients may determine whether bacterial species can successfully colonize specific niches [24, 25]. For example, in the human gut, *S. enterica* manipulates the host cells to alter the local metabolite profile, gaining a competitive metabolic advantage over resident microbiota [26]. Similarly, the metabolite composition of the phyllosphere could potentially impact the colonization capacity of human pathogens in this niche. Distinct metabolic shifts have been observed in human pathogenic bacteria exposed to plant tissues from different organs and cultivars [22, 27–29]. The epiphytic persistence of *S. enterica* correlates with its growth in fruit exudates from various tomato cultivars with distinct surface metabolomic profiles [30]. Likewise, isolate- and plant-specific growth capacities have been reported for pathogenic *E. coli* in apoplastic wash fluid (AWF) recovered from lettuce and spinach leaves [31, 32]. These findings indicate that preexisting and potentially induced plant metabolomic profiles may play a role in either supporting or restricting bacterial contamination in plant tissues.

In this study, we investigated how *S. enterica* and *E. coli* O157:H7 contamination of the leaf apoplast alters transcriptomic and metabolomic profiles of lettuce cultivars with a contrasting bacterial survival phenotype. Integration of these omics’ datasets revealed key cultivar-specific responses that may influence pathogen survival, providing novel insights to inform strategies toward enhanced safety of fresh produce.

## Results

### O157:H7 and STm 14028s differentially modulate the transcriptome of lettuce cultivars

Previously, we have determined that growth of *E. coli* O157:H7 (hereafter O157:H7) and *S. enterica* serovar Typhimurium (STm) 14028s varies in different cultivars of lettuce [19]. Thus, we chose three cultivars, Green Towers (GT), Lollo Rossa (LR), and Red Tide (RT) (Fig. S1), which showed a contrasting bacterium survival trait to gain deeper insights into the metabolic processes altered upon bacterial contamination. To this end, we first analyzed the transcriptome profile of lettuce leaves at 1 and 7 DPI (Dataset S1).

Hierarchical clustering analysis of Log_2_ fold change (FC) values (bacterium- vs. mock-inoculation for each cultivar and time point) derived from normalized read counts aligned to all detected 19,928 lettuce transcripts (Dataset S2) formed two primary clusters: one comprising of all RT treatments and GT at 7 DPI and another consisting of all LR treatments and GT at 1 DPI (Fig. S2). The latter showed more pronounced relative gene expression levels. The second clustering level separated RT at 1 DPI and LR at 1 DPI, respectively. The third clustering level was formed by the day of sampling, in which transcription profiles of each cultivar were closest between both bacterial treatments within the same day (Fig. S2).

Next, we identified significant differentially expressed genes with pair-wise comparisons (bacterial vs. mock inoculations) for each cultivar and time point. LR exhibited the largest changes in the presence of STm 14028s or O157:H7, followed by GT and RT (Table 1). For instance, at 1 DPI, STm 14028s induced significant modulation of 4,448 genes in LR, in contrast to 1,458 in GT and 346 in RT. Moreover, STm 14028s elicited larger changes in gene expression compared to O157:H7 among all lettuce cultivars (Table 1). Overall, transcriptional changes were more substantial at 1 DPI than at 7 DPI, except for O157:H7 in GT and RT where only a few DEGs were detected (Table 1). Intersection analysis revealed the level of overlap among DEGs detected for each pairwise comparison. A minimum overlap was observed within GT and RT samples due to the low number of DEGs, whereas LR samples showed large overlaps (Fig. S3A-C). For instance, 566 downregulated (46.4%) and 568 upregulated (56.2%) genes in LR inoculated with STm 14028s were shared between 7 DPI and 1 DPI, suggesting a lasting plant genotypic response to this bacterium (Fig. S3B). In addition, there was a high overlap between O157:H7 and STm 14028s treatments in LR at 1 DPI, where 358 (78.2%) downregulated DEGs and 1,495 (87.3%) upregulated DEGs were shared between O157:H7 and STm 14028s treated LR samples (Fig. S3B).

**Table 1:** Number of significant differentially expressed genes (DEGs) and differentially accumulated metabolites (DAMs) in the indicated lettuce cultivar at 1- and 7-days post inoculation (DPI) with either STm 14028s or O157:H7 when compared to mock-inoculated plants. The direction of change, up or down, is based on a Log_2_ fold change ≤ − 1 or ≥ 1 and p-value < 0.05. Adjusted p-values were used for DEGs. The number of DEGs with an assigned KEGG ontology (KO) protein ID are shown in the KO column. The number of dams included known and unknown metabolites. Statistical analysis to identify DEGs and dams are shown in dataset S1 and dataset S6, respectively

			Number of DEGs				Number of DAMs		
Cultivar	Bacterium	DPI	Up	Down	Total	KO	Up	Down	Total
Green Towers	O157:H7	1	1	1	2	1	3	9	12
		7	4	5	9	5	4	6	10
	STm 14028s	1	1139	319	1458	542	12	31	43
		7	85	60	145	54	10	7	17
Lollo Rossa	O157:H7	1	1713	458	2171	856	10	0	10
		7	47	27	74	32	0	0	0
	STm 14028s	1	2632	1816	4448	1817	6	9	15
		7	1012	1220	2232	892	9	15	24
Red Tide	O157:H7	1	2	0	2	1	0	4	4
		7	3	3	6	5	3	0	3
	STm 14028s	1	263	83	346	183	2	3	5
		7	0	6	6	5	15	15	30

The intersection analysis to assess the overlap of DEGs among the cultivars in response to the same bacterium showed little to no overlap in O157:H7-treated samples (Fig. S3D), possibly because this bacterium did not modulate GT and RT transcriptomes as extensively as the LR transcriptome (Table 1). By contrast, the intersection analysis for STm 14,028-treated samples revealed a high level of overlap between GT at 1 DPI and LR at 1 and 7 DPI (Fig. S3E). For instance, 164 downregulated (51.4%) and 829 upregulated (72.8%) genes were shared between GT and LR at 1 DPI with STm 14028s (Fig. S3E).

### Lettuce metabolism is altered by O157:H7 and STm 14028s at the transcriptional level

**Fig. 1:**
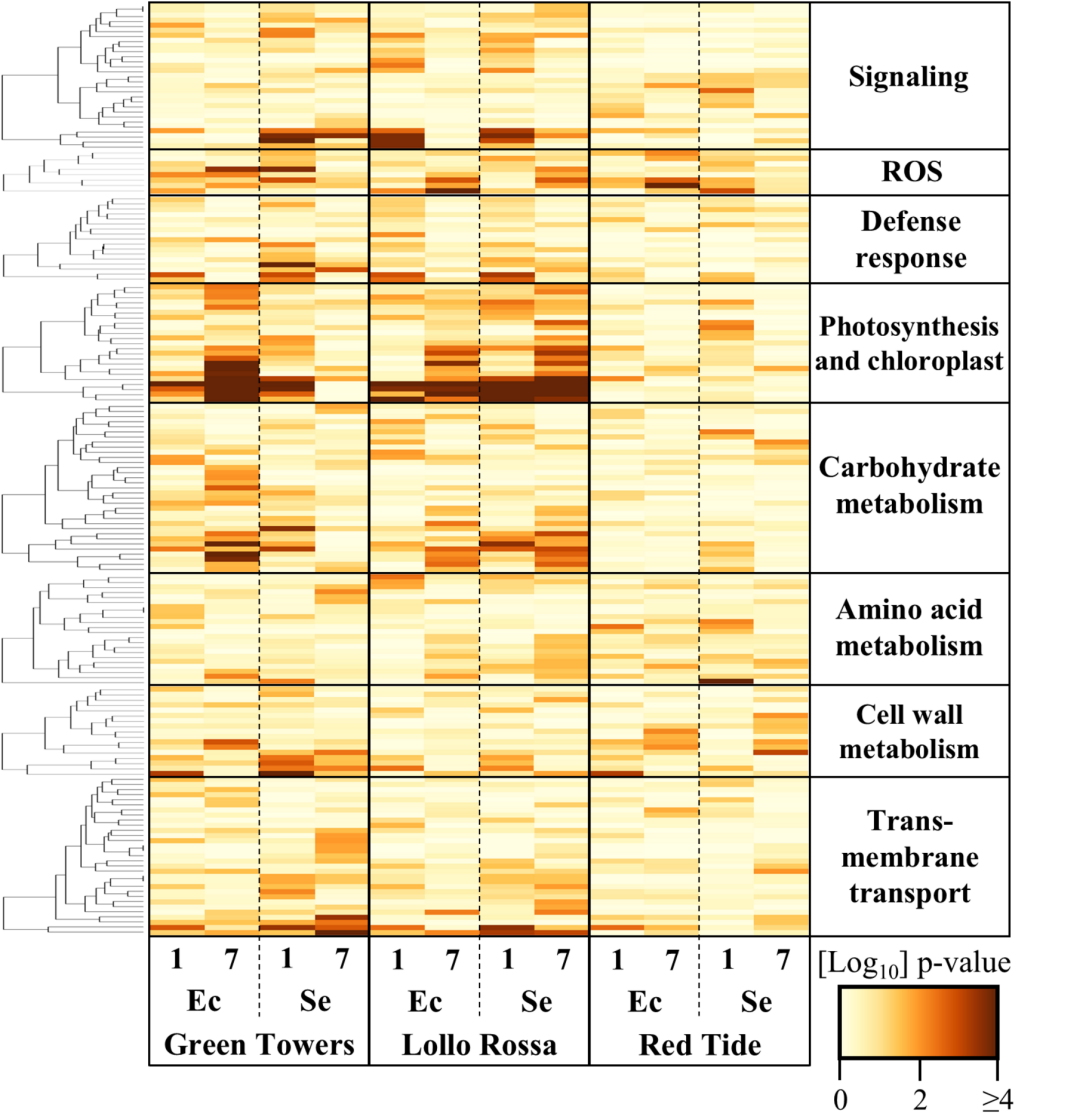
Gene ontology (GO) enrichment analysis of the transcriptomic response of the lettuce cultivars Green Towers, Lollo Rossa, and Red Tide at 1- and 7-days post inoculation with *Escherichia coli* O157:H7 (Ec) or *Salmonella enterica* ser. Typhimurium 14028s (Se). Heat map shows the absolute value of Log_10_*p*-values of significantly (*p* < 0.05) overrepresented GO terms. GO terms are grouped by functional categories listed in Dataset S4. Heatmaps and clustering were conducted using the heatmap.2 R package with default analysis settings. ROS = reactive oxygen species

To identify metabolic processes potentially altered in lettuce cultivars in response to O157:H7 or STm 14028s, we conducted a Gene Ontology (GO) term enrichment analysis (Dataset S3). Significantly enriched (*p* < 0.05) GO terms were grouped according to functional categories, including signaling, reactive oxygen species (ROS), photosynthesis, carbohydrate and amino acid metabolism, cell wall metabolism, and transmembrane transport. The Log_2_ p-values of the enrichment analysis across all treatments are shown in Dataset S4 and were used as input for hierarchical clustering analysis and heatmap visualization (Fig. 1). Within the signaling category, the most significantly enriched GO terms were protein phosphorylation (GO:0006468), protein autophosphorylation (GO:0046777), and cell surface receptor signaling pathway (GO:0007166). These processes were highly regulated in GT by STm 14028s at 1 DPI and in LR by STm 14028s and O157:H7. GO terms related to ethylene, salicylic acid, abscisic acid, and auxin biosynthesis and signaling processes were also significantly overrepresented, particularly in LR and GT. Modulation of the defense responses was also evident, including GO terms associated with systemic acquired resistance (GO:0010112 and GO:0009862), as well as response against bacterium (GO:0050829 and GO:1900426), fungus (GO:0050832) and insect (GO:1900367) were among the most significantly enriched terms. The regulation of these functions by O157:H7 and STm 14028s was particularly high in LR and GT compared to RT. Photosynthesis was distinctly affected, with various GO terms among the most significantly overrepresented in the lettuce cultivars GT and LR upon exposure to O157:H7 and STm 14028s. In contrast, processes linked to photosynthesis were minimally altered in the foliar apoplast of RT after the inoculations (Fig. 1).

**Fig. 2:**
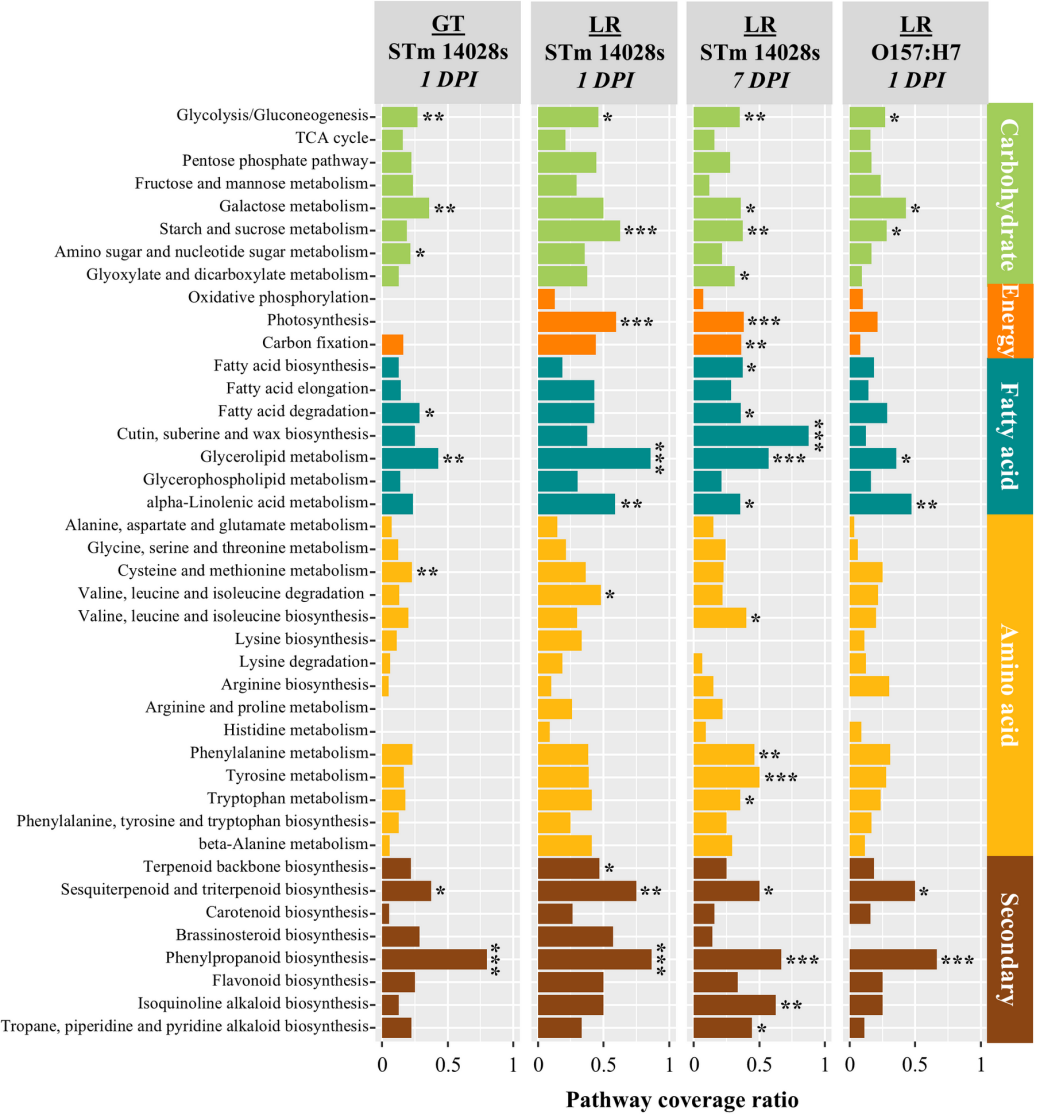
KEGG pathway enrichment analysis of differentially expressed genes (DEGs) in the lettuce cultivars Green Towers (GT) and Lollo Rossa (LR) at 1- and 7-days post inoculation (DPI) with *Salmonella enterica* ser. Typhimurium 14028s or *Escherichia coli* O157:H7. Pathway coverage ratio corresponds to the number of DEGs mapped to a KEGG pathway divided by the total number of genes comprising that KEGG pathway. To map the genes to the metabolic pathways, the protein sequences of the corresponding annotated genome were used to obtain the KEGG protein IDs using the KofamKOALA BLAST tool (https://www.genome.jp/tools/kofamkoala/). Protein sequences with an E-value < 1 × 10^− 5^ (Dataset S1) were used as input for the KEGG Mapper Reconstruct Pathway tool (https://www.kegg.jp/kegg/tool/map_pathway.html). Mapped KEGG metabolic categories are listed on the right. Enrichment analysis was conducted with the hypergeometric test and significantly enriched pathways are marked with *, where * = *p* < 0.5; ** = *p* < 0.01; *** = *p* < 0.001

To gain further insights into the metabolic pathways altered by bacterial contamination as a factor of the plant cultivar, we mapped lettuce transcript sequences to KEGG pathways. Out of the total lettuce transcripts (19,928) captured in the RNA-seq analysis, 9,213 (46.2%) could be assigned to a KEGG Ontology (KO) protein ID, with 3,821 being unique, indicating multiple genes being assigned to the same protein ID (Dataset S1). KO protein IDs were used to reconstruct a reference for the overall metabolic routes represented in the dataset (Fig. S4A). To identify KEGG metabolic routes that were modulated upon bacterium contamination, DEGs with a KO protein ID (Table 1; Dataset S1) were mapped to KEGG pathways (Fig. S4B). Enriched KEGG metabolic pathways were only detected in GT at 1 DPI with STm 14028s, LR at 1 and 7 DPI with STm 14028s, and LR at 1 DPI with O157:H7 (Fig. 2). The relatively low number of DEGs with KO proteins IDs (Table 1; Dataset S1; Fig. S4B) did not allow the identification of pathways in other treatments. Nonetheless, the KEGG pathway enrichment analysis showed 23 metabolic pathways that are either uniquely or commonly overrepresented in those treatments, these pathways fall within the broad categories of carbohydrate, energy, fatty acids, amino acids, and secondary metabolites (Fig. 2).

Carbohydrate metabolism was significantly altered in the lettuce cultivars LR and GT. Enriched KEGG pathways included those related to galactose, glucose, fructose, starch, and sucrose metabolism, while the TCA cycle and pentose phosphate pathway were not significantly affected (Fig. 2). Pathways in the energy metabolism category of KEGG, including photosynthesis and carbon fixation, were also enriched in LR at 1 and 7 DPI with STm 1428s (Fig. 2). Various primary pathways were significantly modulated potentially towards the biosynthesis of secondary metabolites. For instance, the fatty acid degradation pathway was enriched in LR, possibly generating Acetyl-CoA for terpenoid synthesis through the mevalonate pathway (Fig. 2). The metabolism of alpha-linolenic acid, involved in the biosynthesis of jasmonates [33], was also significantly enriched in LR post inoculations with O157:H7 and STm 14028s (Fig. 2). In addition, the KEGG pathway for the degradation of valine, leucine, and isoleucine were significantly enriched in LR at 1 DPI with STm 14028s (Fig. 2). These pathways are connected downstream with the biosynthesis of secondary metabolites such as terpenoids that were significantly stimulated in LR (Fig. 2). Furthermore, the complex phenylpropanoid biosynthetic pathways were substantially altered in LR and GT at 1 DPI with STm 14028s (Fig. 2).

Overall, transcriptome profiling revealed that the response to O157:H7 and STm 14028s varied among lettuce cultivars, with LR and GT exhibiting more similar and stronger regulation of the immune system and metabolic processes than RT.

### The apoplastic metabolome is differentially modulated by O157:H7 and STm 14028s

To investigate the chemical changes in the apoplast of lettuce leaves colonized by STm 14028s or O157:H7, we extracted AWF from leaves of the three lettuce cultivars and analyzed their metabolome profile. A total of 332 metabolites were detected and quantified based on normalized peak heights (Dataset S5). Hierarchical clustering analysis of the metabolome profiles revealed clear distinctions among treatments, which primarily clustered according to lettuce genotypes, indicating cultivar-specific metabolome and responses to the inoculation treatments (Fig. S5). The analysis also showed a temporal impact on metabolite relative abundance, with some compounds maintaining consistent levels while others fluctuating over time. Within these primary groups, inoculation type further contributed to the variation. All STm 14028s-treated samples consistently separated from mock-treated samples for both time points, suggesting a strong and sustained metabolic modulation (Fig. S5). The clustering of O157:H7-inoculated samples happened with either STm 14028s- or mock-treated samples, where distinct metabolic differences from the mock treatments were observed in GT at 7 DPI and in LR and RT at 1 DPI (Fig. S5).

**Fig. 3:**
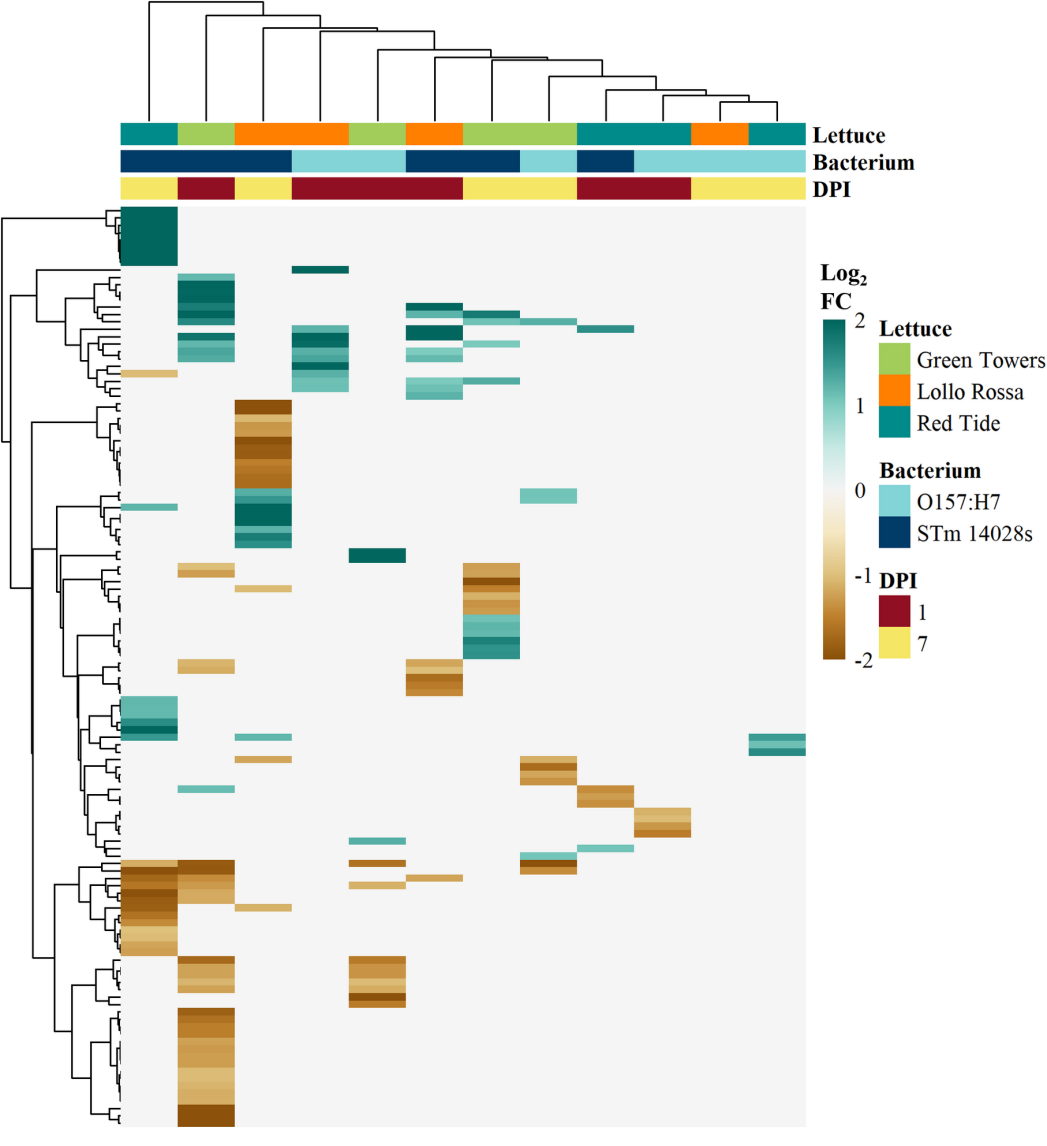
Hierarchical clustering analysis of all 124 known and unknown differentially accumulated metabolites (DAMs) detected in the apoplast wash fluid collected from the lettuce cultivars Green Towers, Lollo Rossa, or Red Tide at 1- or 7-days post inoculation with *Escherichia coli* O157:H7 or *Salmonella enterica* ser. Typhimurium 14028s. Raw peak heights were normalized with Log_10_ transformation and auto-scaling functions and subjected to Student´s *t*-test pair-wise comparisons (bacterium vs. mock) to identify DAMs through the MetaboAnalyst5.0 software. DAMs were determined as those having a p-value < 0.05 and a Log_2_ fold change (FC) ≤ − 1 or ≥ 1. White boxes represent not significant net accumulation. Input data are listed in Dataset S6. Heatmaps and clustering were created with the pheatmap R package using default settings

To assess changes in the metabolome profile upon bacterial contamination, we identified differentially accumulated metabolites (DAMs), defined as those with a Log_2_ FC (bacterium vs. mock) ≤ − 1 or ≥ 1 and p-value < 0.05 (Dataset S6; Table 1). Out of the 332 detected metabolites, 114 have known functions that include 32 sugars and sugar alcohols, 20 amino acids, 32 organic acids, and 7 phenolics (Dataset S6). A total of 124 compounds showed significant net changes in at least one treatment, and DAMs ranged from 0 to 43 depending on the lettuce cultivar and time after inoculation (Dataset S6; Table 1). The hierarchical clustering of treatments based on Log_2_ FC values for all DAMs revealed very distinct metabolite accumulation patterns among lettuce genotypes in response to STm 14028s and O157:H7. Overall, STm 14028s induced more extensive metabolite changes, with GT showing major shifts as early as 1 DPI, while RT and LR exhibited stronger responses at 7 DPI (Fig. 3; Table 1).

**Fig. 4:**
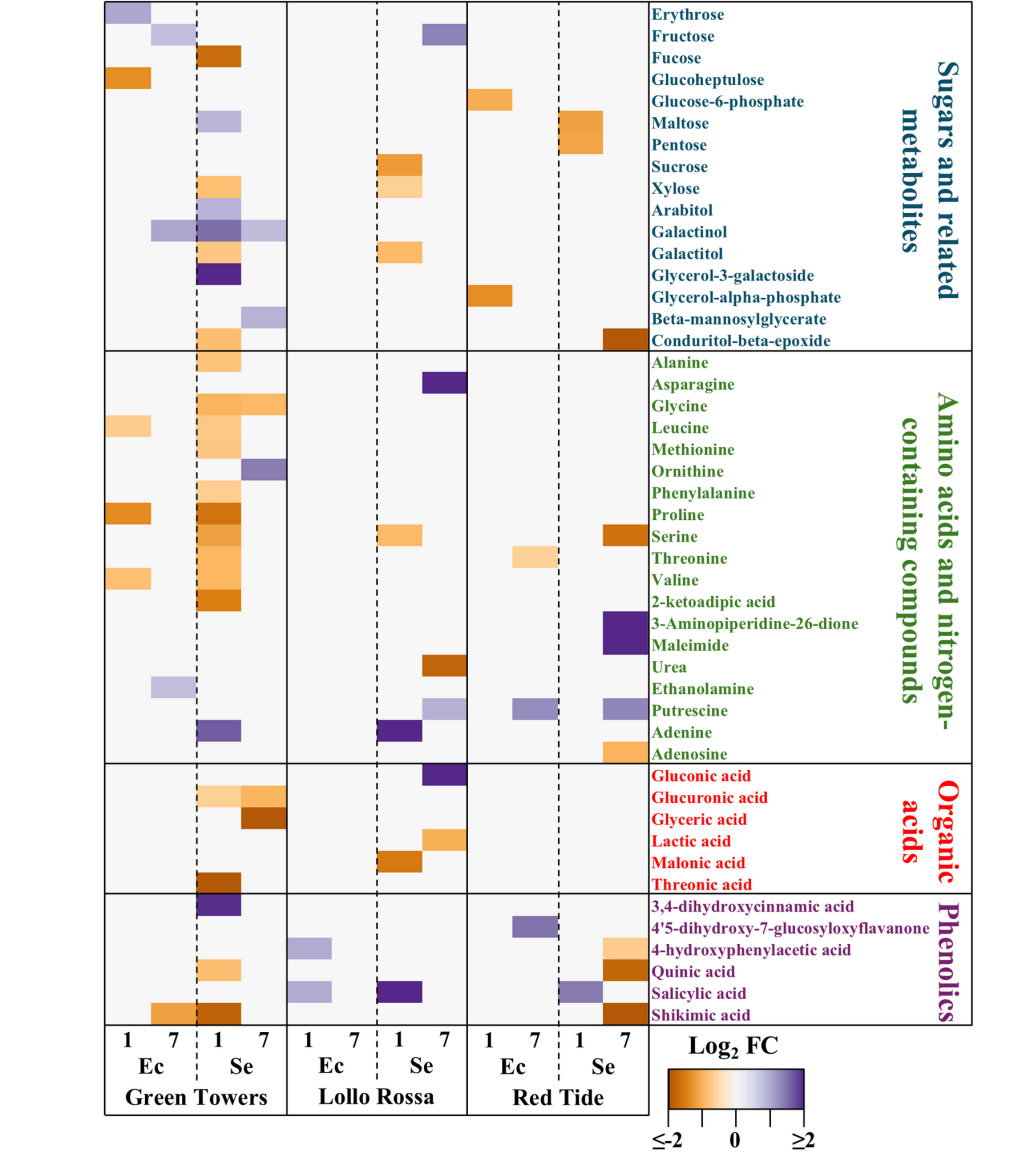
Primary and secondary metabolites with significant differential accumulation in the apoplastic wash fluid collected from the lettuce genotypes Green Towers (GT), Lollo Rossa (LR), and Red Tide (RT) at 1- and 7-days post inoculation with *Escherichia coli* O157:H7 (Ec) or *Salmonella enterica* ser. Typhimurium 14028s (Se). Raw peak heights were normalized with the Log_10_ transformation and auto-scaling functions and subjected to Student´s *t*-test pair-wise comparisons (bacterium vs. mock) to identify differentially accumulated metabolites (DAMs) through the MetaboAnalyst5.0 software. DAMs were determined as those having a *p*-value < 0.05 and a Log_2_ fold change (FC) ≤ − 1 or ≥ 1. White boxes represent not significant net accumulation. Heatmap was created with the heatmap.2 R package using default settings

Next, we created a heatmap to visualize the accumulation dynamics of DAMs with known functional classification identified with all pair-wise comparison (Fig. 4) as follows:

#### Sugars and related metabolites

GT exhibited the most extensive changes in this category. Some metabolites followed similar trends across lettuce genotypes, such as fructose, which accumulated at 7 DPI in GT with O157:H7 and in LR with STm 14028s, and xylose, which decreased in GT and LR at 1 DPI with STm 14028s. In addition, galactitol levels were reduced in LR and GT at 1 DPI following STm 14028s inoculation, while conduritol-beta-epoxide declined in GT and RT under the same conditions. Moreover, galactinol accumulated in GT in response to both pathogens, whereas erythrose and fructose increased exclusively in GT after O157:H7 inoculation. In contrast, RT primarily exhibited significant metabolite reductions in this category.

#### Amino acid and nitrogen-containing compounds

This category displayed particularly pronounced alterations in GT, where STm 14028s triggered a significant decrease in nine amino acids at 1 DPI, including valine, leucine, and proline, which were also reduced in response to O157:H7. Conversely, LR and RT showed fewer and more specific changes. For instance, maleimide levels increased in RT at 7 DPI with STm 14028s, while adenine accumulation was observed in LR at 1 DPI with STm 14028s.

#### Organic acids

These compounds were only differentially accumulated in GT and LR. A significant decline of glucuronic acid, glyceric acid, and threonic acid was induced by STm 14028s in GT. In LR, gluconic acid levels increased significantly following STm 14028s inoculation, whereas lactic acid and malonic acid levels declined.

#### Secondary metabolites

Six phenolic compounds were differentially accumulated across treatments. The phenolic acid precursors quinic acid and shikimic acid decreased in GT and RT following STm 14028s inoculation, with GT displaying an earlier response. Additionally, 3,4-dihydroxycinnamic acid (caffeic acid) significantly increased in GT at 1 DPI with STm 14028s, whereas 4-hydroxyphenylacetic acid accumulated in LR and 4’5-dihydroxy-7-glucosyloxyflavanone in RT following O157:H7 inoculation. Salicylic acid (SA) significantly increased in LR and RT at 1 DPI with STm 14028s but remained unchanged in GT. Moreover, LR also exhibited SA accumulation at 1 DPI in response to O157:H7.

### GT and LR show variable levels of SA-dependent immune responses

Our multi-omics analysis revealed that lettuce cultivars LR and GT underwent substantial transcriptomic reprogramming, particularly through the activation of defense-related genes (Fig. 2), alongside distinct metabolite accumulation patterns in the apoplast (Fig. 4). Notably, LR exhibited a stronger overall response than GT (Table 1) and significantly accumulated SA (Fig. 4) in the presence of bacteria. These findings prompted us to test whether these bacteria induce hallmark SA-dependent defense responses in our cultivars.

**Fig. 5:**
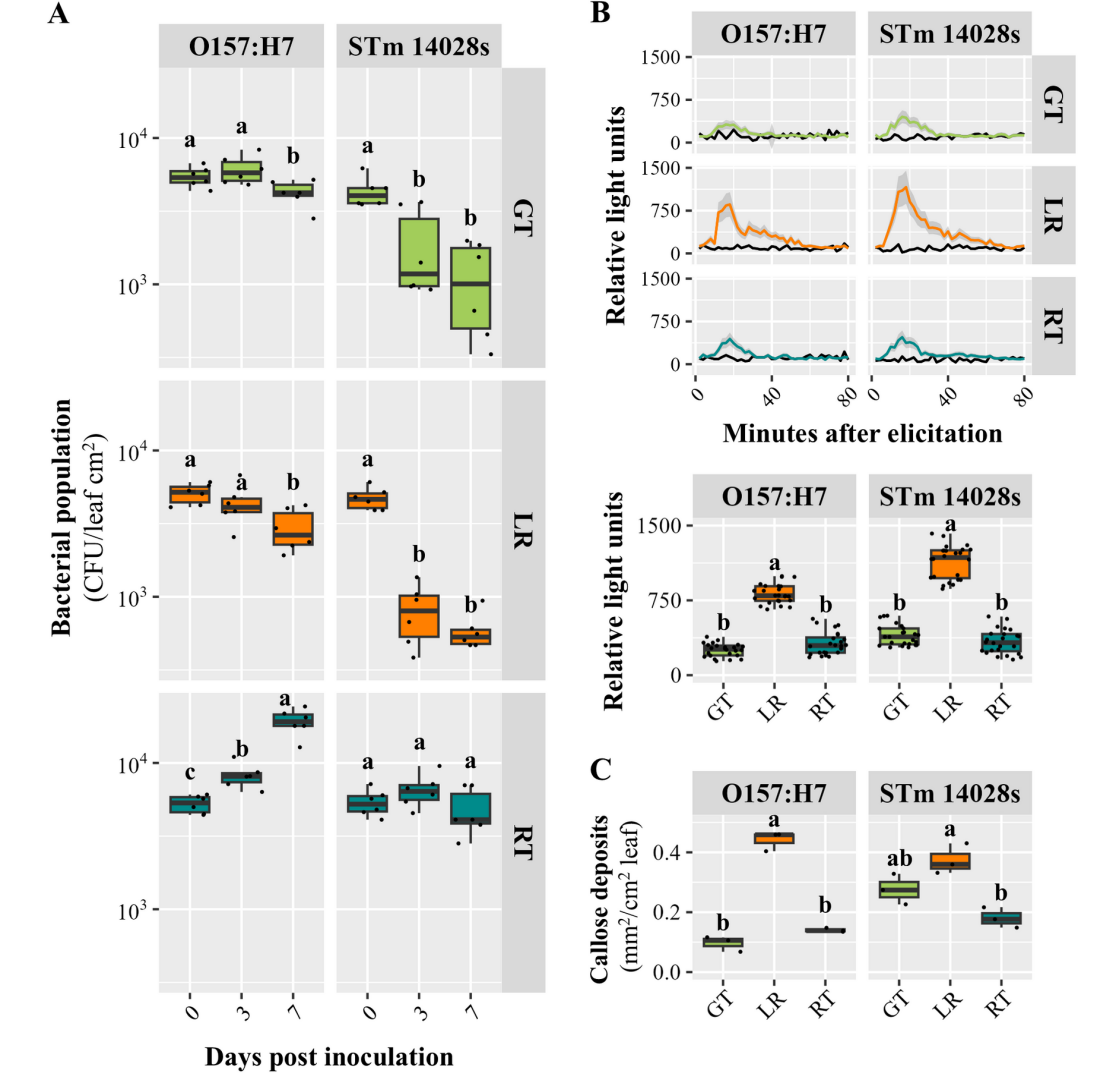
GT and LR show similar bacterial population declined, but variable levels of SA-dependent immune responses. (**A**) *Escherichia coli* O157:H7 and *Salmonella enterica* ser. Typhimurium 14028s population kinetics in leaves of Green Towers (GT), Lollo Rossa (LR), and Red Tide (RT). Apoplastic bacterial population size was estimated by serial dilution plating method using surface sterilized leaves collected at 0-, 3- and 7-days post inoculation. Three plants were used for each sampling point and graphs show combined data from two independent experiments (*n* = 6). (**B**) Temporal production of reactive oxygen species after elicitation with mock (black lines) or bacterial solutions. The curve peak (16–18 min after elicitation) was used to assess statistically significant differences among the genotypes. (**C**) Callose deposition was quantified as the area covered by deposits (mm^2^) over cm^2^ leaf at 24 h after inoculations Graphs in **B** and **C** show the peak value (*n* = 24) or callose deposition area (*n* = 3), respectively, of bacterium-treated samples normalized by the mock-treated samples. Different letters on the top of adjacent boxes (*i*.*e*., within the plant genotype or days post inoculation) indicate significant statistical differences among the means calculated with ANOVA followed by Tukey’s test (α = 0.05)

First, we confirmed that O157:H7 and STm 14028s populations significantly decreased (*p* < 0.001) in the leaf apoplast of GT and LR at 7 DPI, whereas O157:H7 titters increased (*p* < 0.0001) in RT leaves, and STm 14028s remained unchanged (*p* = 0.064) (Fig. 5A). Then, we evaluated ROS burst and callose deposition in leaves after bacterial inoculations as described by Jacob and Melotto [19]. Interestingly, O157:H7- and STm 14028s-induced ROS burst in GT leaves were comparable to that in RT, however significantly lower (*p* < 0.0001) than in LR (Fig. 5B). Likewise, O157:H7 induced similar levels of callose deposits in GT and RT, both significantly lower (*p* < 0.0001) than in LR, while STm 14028s provoked an intermediate callose response in GT, between LR and RT (Fig. 5C).

Overall, these findings suggest that the survival of these human bacterial pathogens in the lettuce cultivar GT is only partially explained by SA-dependent plant immune responses.

## Discussion

Bacterial pathogens of human can reach the leaf interior through stomatal pores [34–39]. Once in the apoplast, these pathogens might encounter a more favorable environment than the exposed leaf surface [40, 41] and gain protection from standard sanitization practices used in the leafy greens industry [42–44]. However, their survival in this niche is strongly influenced by plant immune responses [19, 45, 46]. Furthermore, it is expected that the fate of human pathogens in the apoplast is further shaped by specific plant-microbe-microbe interactions [47, 48], environmental conditions [49, 50], and bacterial traits [23, 51]. By integrating temporal leaf transcriptome and leaf apoplast metabolome analyses, we focused on identifying plant molecular components that may contribute to reduced bacterial survival in distinct lettuce cultivars.

Our findings provided evidence that LR might rely on SA-dependent defense mechanisms to counter STm 14028s and O157:H7 colonization [18, 52]. The significant apoplastic accumulation of SA (Fig. 4) coincided with the upregulation of genes involved in SA biosynthesis (Dataset S1), including *EPS1*, *arogenate dehydratase*, and *phenylalanine ammonia-lyase* [53, 54]. LR also exhibited a stronger ROS burst (Fig. 5B) associated with the induction of *OXI1* and *RBOH* genes that are key regulators of ROS-mediated defense [55, 56]. Interestingly, it has been reported that increase in H_2_O_2_ concentration is associated with O157:H7 population decrease in the lysate of middle and young leaves of different lettuce types [57]. Furthermore, increased callose deposition (Fig. 5C) corresponded with the upregulation of *CALLOSE SYNTHASE* genes in response to both bacteria (Dataset S1), suggesting robust cell wall reinforcement [58]. The upregulation of *EXO70* homologs, especially in response to STm 14028s (Dataset S1), further indicate active vesicle trafficking mechanisms involved in human pathogen restriction [45]. Thus, these findings above align with previous reports demonstrating the importance of SA signaling in limiting STm 14028s and O157:H7 colonization in plants [45, 46, 59].

We observed that GT might have distinct defense mechanisms, differing significantly from LR despite commonalities in transcriptomic responses (Figs. 2 and 3, S2, S3). This genotype exhibited net accumulation of apoplastic caffeic acid and upregulation of *shikimate O-hydroxycinnamoyltransferase* gene, suggesting a role for hydroxycinnamic acid metabolism in its defense strategy [60, 61]. Although caffeic acid biosynthesis has been linked to SA-dependent defense responses [62], GT did not show increased apoplastic SA (Fig. 4) and partially induced PTI-associated defenses (Fig. 5). Interestingly, GT activated ethylene biosynthesis and signaling pathways, including the upregulation of genes encoding SAM synthetase, ACC synthase, ACC oxidase, and multiple ethylene-responsive transcription factors (Dataset S1), supporting the role of this hormone in defense against human pathogens [46, 59, 63]. GT also accumulated galactinol after exposure to both pathogens, a signaling metabolite implicated in biotic stress mitigation [64, 65]. Furthermore, GT exhibited a significant reduction in the accumulation of nine amino acids in the apoplast (Fig. 4). The availability of nutrients in this foliar environment can significantly impact the survival of human bacterial pathogens [31, 32]. These findings suggest that, while GT exhibits a less pronounced defense response than LR, it has distinct mechanisms to restrict STm 14028s and O157:H7 persistence.

Compared to LR and GT, RT exhibited surprisingly marginal transcriptional and metabolic modulation, with minimal activation of PTI-associated defenses in response to both STm 14028s and O157:H7. While a few genes were differentially expressed in response to STm 14028s at 1 DPI, most metabolite changes in the apoplast occurred later at 7 DPI (Table 1). This weak response may have played a role in limiting STm 14028s population growth, nonetheless it persisted over time (Fig. 5). In contrast, the apparent lack of an effective defense response in RT likely facilitated a significant increase in the O157:H7 apoplastic population.

We have identified some molecular components involved in the differential response of lettuce genotypes to STm 14028s and O157:H7 among water-soluble and primary metabolites (Dataset S5). However, proteins, secondary metabolites, and ions could also influence the differential apoplastic interaction between lettuce and human pathogens [8, 66]. In addition, metabolite dynamics in this niche might not be solely plant-driven, where both plant and bacterial responses may contribute to the chemical composition of contaminated leaves. Furthermore, the contribution of post-transcriptional modulation of biosynthetic pathways and metabolite transport should not be overlooked. Future studies in this direction should enhance the understanding of how these additional factors might have an impact on the observed phenotypes.

Overall, STm 14028s elicited a stronger response than O157:H7 at transcriptomic, metabolomic, and phenotypic levels. Furthermore, O157:H7 exhibited higher survival across the lettuce genotypes (Fig. 5), aligning with our previous findings with various lettuce genotypes as well as Arabidopsis [19, 22, 67]. The molecular mechanisms underlying differential activation of plant immunity by various human pathogenic bacteria are not fully understood. However, variations in bacterial extracellular appendages recognized by plant receptors [21] and the ability to suppress plant defenses [51, 68] have been proposed as contributing factors.

## Conclusion

This study provides evidence that lettuce cultivars exhibit distinct molecular responses that could influence the survival of human bacterial pathogens in the apoplast. Depending on the cultivar, immune activation or metabolite composition may have a prominent role in restricting human pathogen growth in the leaf apoplast. These findings offer novel insights into the genetic and biochemical factors shaping lettuce-pathogen interactions, which could inform breeding programs and agronomic practices aimed at enhancing food safety.

## Methods

### Plant material and growth conditions

The loose-leaf lettuce (*Lactuca sativa* L. var. *acephala* Dill.) cultivars Lollo Rossa (LR) and Red Tide (RT) and the romaine lettuce (*L*. *sativa* L. var. *longifolia* (Lam.) Janchen) cultivar Green Towers (GT) were grown as previously described [19]. Briefly, lettuce plants were grown in pots containing a commercial soil mix (Sun Gro^®^ Sunshine^®^ #1 Grower Mix with RESiLIENCE™, MA, USA), under photosynthetically active light intensity of 240 ± 10 µmol/m^2^/sec with a 12-hour photoperiod, and day and night conditions of 19 ± 1 °C and 75 ± 4% relative humidity (RH) and 18 ± 1 °C and 92 ± 2% RH, respectively. All inoculations were conducted with 3.5- to 4-week-old plants (Fig. S1).

### Bacterial strains and inoculum Preparation

The non-typhoid *S. enterica* subspecies *enterica* serovar Typhimurium strain 14028s [69] and the enterohemorrhagic *E. coli* serotype O157:H7 strain 86 − 24 [70] were grown in Low Salt Luria-Bertani (LSLB) medium (10 g/L tryptone, 5 g/L yeast extract, 5 g/L NaCl, pH 7.0) at 28 °C. Medium was supplemented with 50 µg/mL of streptomycin to grow O157:H7. To prepare the inoculum, the bacterial culture was obtained by streaking cells from frozen glycerol stocks onto solid LSLB medium and incubated overnight. From this culture, a single colony was used to inoculate liquid LSLB medium, which was incubated until reaching an OD_600_ of 0.9 to 1. Bacterial cells were collected by centrifugation at 1,360 x*g* for 20 min at 20 °C (Eppendorf Centrifuge 5810R, Rotor 157 A-4-81, Hamburg, Germany) and suspended in sterile distilled water (SDW) to obtain the desired inoculum concentration.

### Leaf apoplast inoculation and bacterial enumeration

Bacterial cells were vacuum infiltrated into the leaf intercellular space of the lettuce plants as previously described [67]. Briefly, a 5 × 10^5^ CFU/mL inoculum containing 0.01% Silwet^®^-L77 or a mock solution of SDW with 0.01% Silwet^®^-L77 were used for plant inoculations. Inoculated plants were maintained under the same environmental conditions used for plant growth. The second and third leaves of LR and the third and fourth leaves of RT and GT were sampled for bacterial enumeration as they were the two youngest, fully expanded leaves of each lettuce cultivar. At 0-, 3-, and 7-days post inoculation (DPI), the bacterial population was quantified by serial dilution plating as described by Jacob et al. [71]. The leaves were surface sterilized prior to bacteria enumeration by gentle washing in 2% (v/v) sodium hypochlorite for 1 min, 70% (v/v) ethanol for 1 min, and SDW for 1 min, followed by blotting onto a paper towel. To obtain a representative sample of the total leaf area, we collected four 0.5 cm² discs using a cork borer, two from each leaf. The leaf discs were punched from the center of the leaf blade, one on each side of the midvein. Three plants were used per sampling point and the experiment was conducted twice with independent batches of plants (*n* = 6). Bacterial population changes during the experiment were analyzed using ANOVA (aov function), and differences among DPI means were assessed by Tukey’s test (HSD.test function; α = 0.05) in R [72].

### Transcriptomic profiling of lettuce leaves

At 1 and 7 DPI with each bacterium or mock solution, two leaves of each plant (*n* = 3) were excised at the base and quickly processed for analyses. A piece of foliar tissue was used for bacterial population enumeration, and the rest were placed into a 2 mL tube and immediately frozen in liquid nitrogen. Tissue disruption, RNA extraction, and RNA quality assessment were carried out as described in detail by Jacob et al. [22]. High quality RNA was used for library preparation and sequencing (Illumina HiSeq 4000) by the DNA Technologies & Expression Analysis Core Laboratory at the University of California (UC), Davis, USA following a standard protocol for 3′ tag-sequencing. Reads were analyzed by the UC Davis Bioinformatics Core. Briefly, quality control and processing (*i*.*e*., screening, trimming, and filtering) of reads were performed with the HTStream pipeline (https://s4hts.github.io/HTStream/). Alignment of processed reads to the lettuce genome (Lsat_Salinas_v7; accession GCA_002870075.1) and gene counts were conducted with the STAR software (https://github.com/alexdobin/STAR).

Differential gene expression analysis was performed using the edgeR package in R. Counts were normalized (counts per million), followed by Voom transformation and Limma linear model fitting. Group contrasts were analyzed using eBayes function, and differentially expressed genes (DEGs) were determined as those having an adjusted p-value < 0.05 and a Log_2_ fold change ≤ − 1 or ≥ 1. The relationship among replicates of the whole leaf RNA-seq experiment was assessed through multidimensional scale (MDS) plotting (Fig. S6A). Coordinates for MDS graphs were calculated using normalized read counts in the cmdscale function in R. Hierarchical clustering analysis of the transcriptomic dataset was performed with the pheatmap package in R with default settings. Unique and common DEGs among the samples were determined with the UpSetR function in R [73]. The enrichment analysis for gene ontology (GO) was conducted using the Kolmogorov-Smirnov test in the topGO package in R [74]. Metabolic pathway reconstruction was carried out through the KEGG Mapper tool (https://www.kegg.jp/kegg/mapper/reconstruct.html) [75], as previously described [22]. Briefly, lettuce protein sequences (Lsat_Salinas_v7; GCA_002870075.1) were analyzed in the KofamKOALA tool (https://www.genome.jp/tools/kofamkoala/) [76] to obtain KEGG protein IDs that were mapped in KEGG Mapper. Enrichment analysis of KEGG pathways was conducted through the hypergeometric test for over-representation of success as previously described [77].

### Metabolomic profiling of apoplastic wash fluid (AWF)

#### Collection of AWF and quality control

Water-soluble metabolites present in the leaf apoplast of the different lettuce cultivars were recovered by extracting AWF following a previously described infiltration-centrifugation method [23, 78]. At 1 and 7 DPI, leaves were cut and immediately rinsed with SDW for 1 min to remove leaf surface contaminants. Leaves were carefully rolled and placed into a 60 mL syringe filled with SDW. The syringe plunger was pulled to generate negative pressure and gently relaxed to fully infiltrate the lettuce leaves. Water on the leaf surface was carefully removed with paper towel and leaves were rolled around 1 mL pipet tips, wrapped with parafilm, and put into a 50 ml centrifuge tube. Tubes were centrifuged for 8 min at 4 °C to collect the AWF, which was filter-sterilized through a 0.22 μm syringe filter (Restek, PA, USA), frozen in liquid nitrogen, and stored at -80 °C. Each replicate represents AWF collected from two leaves of each plant and four plants were used for each sampling point (*n* = 4).

The centrifugation force (x*g*) required to extract AWF effectively can vary widely across plant species, ranging from 6,000 x*g* in rice [79] to 200 x*g* in spinach [31]. Due to variations in the leaf morphology of our lettuce cultivars, we optimized AWF recovery from each of them while minimizing cytoplasmic contamination. The volume of extracted AWF increased with stronger centrifugation forces (Fig. S7A). However, the electrical conductivity (EC) of the AWF, measured using an EC meter (LAQUAtwin-EC-11, Horiba, Kyoto, Japan), also increased with stronger centrifugation (Fig. S7B), suggesting reduced membrane integrity and potential cytosolic contamination. The optimal centrifugation force that yielded sufficient volume of AWF for downstream experiments was set at 660 x*g* for GT, 532 x*g* for LR, and 299 x*g* for RT. Furthermore, the cellular contamination of the AWF samples was measured with the cytoplasmic enzyme glucose-6-phosphate dehydrogenase (G6PD) assay kit (MAK015-1KT, Sigma-Aldrich, MO, USA) following the manufacturer’s protocol (Fig. S8). Only AWF samples showing enzymatic activity comparable to the negative control were used for the experiments. The negative control corresponded to the zero-concentration NADH standard solution provided with the kit.

#### Untargeted metabolite profiling analysis

The detection and quantification of the metabolites present in the AWF obtained at 1 and 7 DPI was performed by gas chromatography time-of-flight mass spectrometry (GC-TOF-MS) analysis at the UC Davis West Coast Metabolomics Center following previously described procedures [80–82]. Briefly, samples (0.5 µl) were injected into an Agilent 6890 gas chromatograph (Santa Clara, CA, USA) and a Leco Pegasus IV time of flight spectrometer controlled by Leco ChromaTOF software vs. 2.32 (St Joseph, MI, USA) for data pre-processing. Resulting data files with peak heights were exported and further processed by a filtering algorithm implemented in the metabolomics BinBase database [82]. To account for differences in machine sensitivity, tuning, maintenance status, and other parameters, raw data was normalized to the sum of all identified metabolites of the samples [80].

#### Data processing

The metabolomic dataset was analyzed with the MetaboAnalyst5.0 software (https://www.metaboanalyst.ca/) [83]. Metabolite peak heights were normalized by Log_10_ transformation and auto scaling (mean-centered and divided by the standard deviation of each variable) functions. Normalized data was used to determine the relationship between replicates through principal component analysis (PCA) plotting (Fig. S6B). Student´s *t*-test was used to assess the effect of the treatment (mock vs. bacterial inoculations) on the relative abundance of metabolites and to identify differentially accumulated metabolites (DAMs), which were determined as those having a p-value < 0.05 and a Log_2_ fold change ≤ − 1 or ≥ 1. Hierarchical clustering analysis of the metabolomic dataset was performed with the pheatmap package in R with default settings.

### Plant immune response assays

Two hallmark plant immune responses, reactive oxygen species (ROS) burst and callose deposition, were assessed as described in Jacob and Melotto [19]. ROS burst response was measured through a bioassay using 0.2 cm^2^ leaf discs from GT, LR, and RT. The discs were placed individually into wells of a 96-well microplate containing 200 µL of SDW and incubated overnight at constant light and 22 °C to reduce the wounding response. After incubation, SDW was replaced with 100 µL of the elicitation solution composed of 5.38 units of horseradish peroxidase (MilliporeSigma, Burlington, MA, USA) and 34 µg of luminol (MilliporeSigma, Burlington, MA, USA) per mL of SDW with or without 5 × 10^8^ CFU/mL of either O157:H7 or STm 14028s. The elicitation solution containing bacteria was prepared with heat-killed bacterial suspensions (incubated at 100 °C for 10 min) to avoid possible inhibition of ROS production by any unknown virulence factor produced by live bacteria in contact with leaf tissue. After adding the elicitation solution to the wells, plates were immediately inserted into a microplate reader (Synergy H1 Hybrid Multi-Mode Reader, Biotek, Winooski, VT, USA) to measure luminescence and estimate ROS production every 2 minutes between 0 and 90 min. For each treatment, 24 leaf discs were collected from six different plants. The experiment was repeated five times with independent batches of plants.

To quantify callose deposition, leaves were syringe-infiltrated with either water (mock treatment) or 1 × 10^8^ CFU/mL of bacterial inoculum. After 24 hours, the leaf chlorophyll was cleared by immersing leaves into 95% (v/v) ethanol and kept at 37°C for 24 hours on a rotary shaker. Cleared leaves were rinsed in 50% (v/v) ethanol for 1 min, SDW for 1 min twice, 50 mM K_2_HPO_4_ for 3 min, followed by a 1-hour incubation in a 150 mM K_2_HPO_4_ SDW based solution containing 0.05% aniline blue. Leaves were imaged with a Nikon Eclipse 80i fluorescent microscope (Nikon Corporations, Shinagawaku, Tokyo, Japan) equipped with a DAPI (4’,6-diamidino-2-phenylindole) filter, and the NIS Elements Imaging Software Version 4.13.04 was used to process the images. Three leaves of each lettuce cultivar were used per treatment and six images were randomly captured from each side of the midrib (12 pictures per leaf). Infiltrated zones, damaged areas, mid veins, and leaf edges were avoided for imaging to prevent false positive results. The total area of callose deposits (mm^2^ per cm^2^ of leaf) was quantified using the binary tool of the abovementioned software. The experiment was repeated four times with independent batches of plants.

The effect of lettuce genotype on the defense response was analyzed using ANOVA and differences among means for ROS burst peak and callose deposits were assessed with Tukey’s test (α = 0.05).

## Electronic supplementary material

Below is the link to the electronic supplementary material.

**Supplementary Material 1: Fig. S1.** Photos of representative 4-week-old plants of the lettuce cultivars Green Towers (**A**), Lollo Rossa (**B**), and Red Tide (**C**) used in the experiments.

**Supplementary Material 2: Dataset S1. **Gene annotation and corresponding protein KEGG assignment, relative gene expression, and pairwise comparisons for each lettuce cultivar. FC = fold change. DPI = days post inoculation. O157:H7 = *Escherichia coli* O157:H7. STm 14028s = *Salmonella enterica* serovar Typhimurium 14028s.

**Supplementary Material 3: Dataset S2. **Relative expression of all identified transcripts calculated as Log2 fold change between bacterium vs. mock inoculated samples. These values were used to create Figure S2. RT = Red Tide. GT = Green Tower. LR = Lollo Rossa. DPI = days post inoculation. O157:H7 = *Escherichia coli* O157:H7. STm 14028s = *Salmonella enterica* serovar Typhimurium 14028s.

**Supplementary Material 4: Fig. S2. **Hierarchical clustering analysis of all detected transcripts based on Log_2_ fold change (FC) gene expression (bacterium vs. mock) for each lettuce cultivar and time point. Relative gene expression in Green Towers (GT), Lollo Rossa (LR), and Red Tide (RT) at 1- and 7-days post inoculation with *Escherichia coli* O157:H7 (Ec) or *Salmonella enterica* ser. Typhimurium 14028s (Se) is listed in Dataset S2. Heatmap and clustering were conducted using the pheatmap R package with default analysis settings.

**Supplementary Material 5: Fig. S3. **Intersection analysis of differentially expressed genes (DEGs) in the lettuce cultivars Green Towers (GT), Lollo Rossa (LR), and Red Tide (RT) at 1- and 7-days post inoculation (DPI) with *Escherichia coli* O157:H7 or *Salmonella enterica* ser. Typhimurium 14028s. Plots show the number of unique and common DEGs among the treatments for each cultivar (A-C) or for each bacterium (D, E) (X-axis) and eight (A-C) or ten (D, E) intersections exhibiting the highest number of DEGs (Y-axis) for both up and down regulated DEGs. Intersection analyses and plots were generated by using the UpSetR function in R.

**Supplementary Material 6: Dataset S3. **Enrichment analysis of gene ontology (GO) terms within DEG sets identified between pairwise comparisons (bacterium vs. mock) for each cultivar and time point. Significantly enriched (p-value < 0.05) GO terms are highlighted in green. DPI=days post inoculation. O157:H7 = *Escherichia coli* O157:H7. STm 14028s = *Salmonella enterica* serovar Typhimurium 14028s.

**Supplementary Material 7: Dataset S4. **Significantly enriched (p < 0.05) gene ontology (GO) terms identified within DEG sets based on pairwise comparisons (bacterium vs. mock) for each genotype and time point. Terms were grouped into biological categories. Values are shown as Log10 p-value, which were used as input to create Figure 1. Ec = *Escherichia coli* O157:H7. Se = *Salmonella enterica* ser. Typhimurium 14028s. 1 = 1 day post inoculation. 7 = 7 days post inoculation.

**Supplementary Material 8: Fig. S4. **Reconstruction of lettuce KEGG metabolic pathways. To map the lettuce genes to KEGG pathways, the protein sequences of the corresponding annotated genome were used to obtain the KEGG protein IDs using the KofamKOALA BLAST tool (https://www.genome.jp/tools/kofamkoala/). Protein sequences with an E-value < 1x10^−5^ (Dataset S1) were used for the KEGG Mapper Reconstruct Pathway tool (https://www.kegg.jp/kegg/tool/map_pathway.html). All KEGG IDs were used to create lettuce reference metabolic routes (**A**), while the KEGG IDs of significantly differentially expressed genes (adjusted p-value < 0.05 and a Log_2_ fold change ≤ −1 or ≥ 1) were used to identify metabolic pathways modulated in the lettuce cultivars Green Towers (GT) and Lollo Rossa (LR) at 1- and 7-days post inoculation (DPI) with *Salmonella enterica* ser. Typhimurium 14028s or *Escherichia coli* O157:H7 (**B**).

**Supplementary Material 9: Dataset S5. **Normalized peak height values of metabolites detected in the apoplastic wash fluid of the lettuce cultivars Green Tower (GT), Lollo Rossa (LR), and Red Tide (RT) at 1- and 7-days post inoculation (DPI) with mock (MK), Escherichia coli O157:H7 (EC), or *Salmonella enterica* serovar Typhimurium 14028s (SE). Raw peak heights were normalized with the Log10 transformation and auto-scaling functions of MetaboAnalyst5.0 software. These values were used to create hierarchical clustering shown in Supplementary Figure S5.

**Supplementary Material 10: Fig. S5. **Hierarchical clustering analysis of all 332 metabolites detected in the apoplastic wash fluid (AWF) collected from the lettuce cultivars Green Towers, Lollo Rossa, and Red Tide at 1- and 7-days post inoculation with Mock (Mk), *Escherichia coli* O157:H7 or *Salmonella enterica* ser. Typhimurium 14028s. Raw peak heights were normalized with the Log10 transformation and auto-scaling functions of MetaboAnalyst5.0 software. Values are listed in Dataset S5. Heatmaps and clustering were created with the pheatmap R package using default settings.

**Supplementary Material 11: Dataset S6. **Relative accumulation of metabolites detected in the apoplastic wash fluid (AWF) from lettuce leaves inoculated with mock or bacterial inoculum at 1 and 7 days post inoculation (DPI) with *Escherichia coli* O157:H7 or *Salmonella enterica* serovar Typhimurium 14028s. Raw peak heights were normalized with the Log10 transformation and auto-scaling functions and subjected to Student’s t-test pair-wise comparisons (bacterium vs. mock) to identify differentially accumulated metabolites (DAMs) through the MetaboAnalyst5.0 software. DAMs were determined as those having a p-value < 0.05 and a Log2 fold change (FC) ≤ −1 or ≥ 1. DAMs are highlighted in green. FDR = False Discovery Rate.

**Supplementary Material 12: Fig. S6. **Multidimensional scaling (MDS) (**A**) and principal component analysis (PCA) (**B**) plots representing the correlation among biological replicates used for the whole leaf RNA-sequencing and AWF metabolomic analyses, respectively. Leaves of the lettuce cultivars Green Towers, Lollo Rossa, and Red Tide were vacuum infiltrated with mock (sterile distilled water) or bacterial inoculum containing 5 x 10^5^ CFU/mL of *Escherichia coli* O157:H7 or *Salmonella enterica* ser. Typhimurium 14028s. Leaves were sampled at 1- and 7-days post inoculation (DPI). Coordinates for MDS graphs were calculated using normalized read counts in the cmdscale function of the R software and the coordinates for PCA graphs were obtained by using normalized peak heights in the MetaboAnalyst 5.0 software.

**Supplementary Material 13: Fig. S7. **Optimization of the apoplastic wash fluid (AWF) extraction procedure for each lettuce cultivar, Lollo Rossa, Red Tide, or Green Towers. (**A**) Graph shows the volume of infiltrated water (IW) and extracted AWF by gram of leaf tissue. The infiltrated volume of water was calculated by subtracting the initial leaf weight to the weight after infiltration. Pairwise mean comparison (IW versus AWF, for each centrifugation force) was performed with two-tail Student’s *t*-test (ns = not significant; * = p < 0.001; ** = p < 0.0001). (**B**) Graph shows the electric conductivity (EC) of the AWF at different centrifugation forces (x*g*). The effect of different centrifugation forces on the EC of the AWF was assessed through ANOVA followed by Tukey’s test (different letters on top of the boxes indicate statistically differences among the means). For both graphs, a replicate consisted of AWF collected and pooled from four leaves of two plants, and 8 plants were used for each sampling point (n = 4).

**Supplementary Material 14: Fig. S8.** Evaluation of cytoplasmatic contamination of apoplastic wash fluid (AWF) samples based on regression analysis relative to the NADH standard curve. Temporal accumulation (X-axis) of NADH due to the enzymatic activity of cytoplasmic glucose-6-phosphate dehydrogenase in AWF recovered from non-inoculated leaves of the indicated lettuce cultivar. Note that at the end of the assay (20 min) the NADH accumulation in the AFW samples was comparable to the negative control provided with the kit. The plot shows data from two independent experiments (n = 6).

## Data Availability

Raw sequencing data are available at the National Center for Biotechnology Information Short Read Archive, under the BioProject accession code PRJNA1231482. Metabolomic data were deposited in the EMBL-EBI database under the accession number S-BSST1908. All additional data generated or analyzed during this study are included in this published article and its supplementary information files.

## References

[1] Farvardin A, González-Hernández AI, Llorens E, García-Agustín P, Scalschi L, Vicedo B. The Apoplast: a key player in plant survival. Antioxidants. 2020;9:604. 10.3390/antiox9070604. 32664231 10.3390/antiox9070604 PMC7402137

[2] Evans JR, Kaldenhoff R, Genty B, Terashima I. Resistances along the CO_2_ diffusion pathway inside leaves. J Exp Bot. 2009;60:2235–48. 10.1093/jxb/erp117. 19395390 10.1093/jxb/erp117

[3] Gentzel I, Giese L, Zhao W, Alonso AP, Mackey D. A simple method for measuring Apoplast hydration and collecting Apoplast contents. Plant Physiol. 2019;179:1265–72. 10.1104/pp.18.01076. 30824565 10.1104/pp.18.01076 PMC6446764

[4] Sattelmacher B. The Apoplast and its significance for plant mineral nutrition. New Phytol. 2001;149:167–92. 10.1046/j.1469-8137.2001.00034.x. 33874640 10.1046/j.1469-8137.2001.00034.x

[5] Delmer D, Dixon RA, Keegstra K, Mohnen D. The plant cell wall-dynamic, strong, and adaptable-is a natural shapeshifter. Plant Cell. 2024;36:1257–311. 10.1093/plcell/koad325. 38301734 10.1093/plcell/koad325 PMC11062476

[6] Ngou BPM, Ding P, Jones JDG. Thirty years of resistance: Zig-zag through the plant immune system. Plant Cell. 2022;34:1447–78. 10.1093/plcell/koac041. 35167697 10.1093/plcell/koac041 PMC9048904

[7] Borniego ML, Molina MC, Guiamét JJ, Martinez DE. Physiological and proteomic changes in the Apoplast accompany leaf senescence in Arabidopsis. Front Plant Sci. 2020;10:1635. 10.3389/fpls.2019.01635. 31969890 10.3389/fpls.2019.01635 PMC6960232

[8] Dora S, Terrett OM, Sánchez-Rodríguez C. Plant–microbe interactions in the Apoplast: communication at the plant cell wall. Plant Cell. 2022;34:1532–50. 10.1093/plcell/koac040. 35157079 10.1093/plcell/koac040 PMC9048882

[9] Khare E, Mishra J, Arora NK. Multifaceted interactions between endophytes and plant: developments and prospects. Front Microbiol. 2018;9:2732. 10.3389/fmicb.2018.02732. 30498482 10.3389/fmicb.2018.02732 PMC6249440

[10] Olanrewaju OS, Glick BR, Babalola OO. Beyond correlation: Understanding the causal link between Microbiome and plant health. Heliyon. 2024;10:e40517. 10.1016/j.heliyon.2024.e40517. 39669148 10.1016/j.heliyon.2024.e40517 PMC11636107

[11] Zhang Y, Yu X, Zhang W, Lang D, Zhang X, Cui G, Zhang X. Interactions between endophytes and plants: beneficial effect of endophytes to ameliorate biotic and abiotic stresses in plants. J Plant Biol. 2019;62:1–13. 10.1007/s12374-018-0274-5.

[12] Hirt H. Healthy soils for healthy plants for healthy humans: how beneficial microbes in the soil, food and gut are interconnected and how agriculture can contribute to human health. EMBO Rep. 2020;5:e51069. 10.15252/embr.202051069. 10.15252/embr.202051069 PMC7403703 32734701

[13] Osaili TM, Hasan F, Al-Nabulsi AA, Olaimat AN, Ayyash M, Obaid RS, Holley R. A worldwide review of illness outbreaks involving mixed salads/dressings and factors influencing product safety and shelf life. Food Microbiol. 2023;112:104238. 10.1016/j.fm.2023.104238. 36906321 10.1016/j.fm.2023.104238

[14] Yang X, Scharff R. Foodborne illnesses from leafy greens in the united States: attribution, burden, and cost. J Food Prot. 2024;87:100275. 10.1016/j.jfp.2024.100275. 38609013 10.1016/j.jfp.2024.100275

[15] Jones P, Garcia BJ, Furches A, Tuskan GA, Jacobson D. Plant host-associated mechanisms for microbial selection. Front Plant Sci. 2019;10:862. 10.3389/fpls.2019.00862. 31333701 10.3389/fpls.2019.00862 PMC6618679

[16] Aung K, Jiang Y, He SY. The role of water in plant-microbe interactions. Plant J. 2018;93:771–80. 10.1111/tpj.13795. 29205604 10.1111/tpj.13795 PMC5849256

[17] Fatima U, Senthil-Kumar M. Plant and pathogen nutrient acquisition strategies. Front Plant Sci. 2015;6:750. 10.3389/fpls.2015.00750. 26442063 10.3389/fpls.2015.00750 PMC4585253

[18] Yu X, Feng B, He P, Shan L. From chaos to harmony: responses and signaling upon microbial pattern recognition. Annu Rev Phytopathol. 2017;55:109–37. 10.1146/annurev-phyto-080516-035649. 28525309 10.1146/annurev-phyto-080516-035649 PMC6240913

[19] Jacob C, Melotto M. Human pathogen colonization of lettuce dependent upon plant genotype and defense response activation. Front Plant Sci. 2020;10:1769. 10.3389/fpls.2019.01769. 32082340 10.3389/fpls.2019.01769 PMC7002439

[20] Montano J, Rossidivito G, Torreano J, Porwollik S, Sela (Saldinger) S, McClelland M, Melotto M. *Salmonella enterica* serovar typhimurium 14028s genomic regions required for colonization of lettuce leaves. Front Microbiol. 2020;11:6. 10.3389/fmicb.2020.00006. 32038592 10.3389/fmicb.2020.00006 PMC6993584

[21] Garcia AV, Charrier A, Schikora A, Bigeard J, Pateyron S, de Tauzia-Moreau ML, Evrard A, Mithöfer A, Martin-Magniette ML, Virlogeux-Payant I, Hirt H. *Salmonella enterica* Flagellin is recognized via FLS2 and activates PAMP-triggered immunity in Arabidopsis thaliana. Mol Plant. 2014;7:657–74. 10.1093/mp/sst145. 24198231 10.1093/mp/sst145

[22] Jacob C, Velásquez AC, Josh NA, Settles M, He SY, Melotto M. Dual transcriptomic analysis reveals metabolic changes associated with differential persistence of human pathogenic bacteria in leaves of Arabidopsis and lettuce. G3 (Bethesda). 2021;11:jkab331. 10.1093/g3journal/jkab331 10.1093/g3journal/jkab331 PMC8664426 34550367

[23] Jacob C, Student J, Bridges DF, Chu W, Porwollik S, McClelland M, Melotto M. Intraspecies competition among *Salmonella enterica* isolates in the lettuce leaf Apoplast. Front Plant Sci. 2024;15:1302047. 10.3389/fpls.2024.1302047. 38352648 10.3389/fpls.2024.1302047 PMC10861783

[24] Bhagwat A, Haldar T, Kanojiya P, Saroj SD. Bacterial metabolism in the host and its association with virulence. Virulence. 2025;16:2459336. 10.1080/21505594.2025.2459336. 39890585 10.1080/21505594.2025.2459336 PMC11792850

[25] Passalacqua KD, Charbonneau M, O’Riordan MX. Bacterial metabolism shapes the host–pathogen interface. Microbiol Spectr. 2016;4:10. 10.1128/microbiolspec.vmbf-0027-2015. 10.1128/microbiolspec.VMBF-0027-2015 PMC4922512 27337445

[26] Rivera-Chávez F, Bäumler AJ. The pyromaniac inside you: salmonella metabolism in the host gut. Annu Rev Microbiol. 2015;69:31–48. 10.1146/annurev-micro-091014-104108. 26002180 10.1146/annurev-micro-091014-104108

[27] Crozier L, Hedley PE, Morris J, Wagstaff C, Andrews SC, Toth I, Jackson RW, Holden NJ. Whole-transcriptome analysis of verocytotoxigenic *Escherichia coli* O157:H7 (Sakai) suggests plant-species-specific metabolic responses on exposure to spinach and lettuce extracts. Front Microbiol. 2016;7:1088. 10.3389/fmicb.2016.01088. 27462311 10.3389/fmicb.2016.01088 PMC4940412

[28] de Moraes MH, Desai P, Porwollik S, Canals R, Perez DR, Chu W, McClelland M, Teplitski M. *Salmonella* persistence in tomatoes requires a distinct set of metabolic functions identified by transposon insertion sequencing. Appl Environ Microbiol. 2017;83:e03028–16. 10.1128/AEM.03028-16. 28039131 10.1128/AEM.03028-16 PMC5311394

[29] Han M, Schierstaedt J, Duan Y, Nietschke M, Jechalke S, Wolf J, Hensel M, Neumann-Schaal M, Schikora A. *Salmonella enterica* relies on carbon metabolism to adapt to agricultural environments. Front Microbiol. 2023;14:1213016. 10.3389/fmicb.2023.1213016. 37744895 10.3389/fmicb.2023.1213016 PMC10513388

[30] Han S, Micallef SA. Environmental metabolomics of the tomato plant surface provides insights on *Salmonella enterica* colonization. Appl Environ Microbiol. 2016;82:3131–42. 10.1128/AEM.00435-16. 26994076 10.1128/AEM.00435-16 PMC4959065

[31] Merget B, Forbes KJ, Brennan F, McAteer S, Shepherd T, Strachan NJC, Holden NJ. Influence of plant species, tissue type, and temperature on the capacity of Shiga-toxigenic *Escherichia coli* to colonize, grow, and be internalized by plants. Appl Environ Microbiol. 2019;85:e00123–19. 10.1128/AEM.00123-19. 30902860 10.1128/AEM.00123-19 PMC6532046

[32] Merget B, Dobrindt U, Forbes KJ, Strachan NJC, Brennan F, Holden NJ. Variability in growth responses of non-O157 EHEC isolates in leafy vegetables, sprouted seeds and soil extracts occurs at the isolate level. FEMS Microbiol Lett. 2020;367:fnaa030. 10.1093/femsle/fnaa030. 32068797 10.1093/femsle/fnaa030

[33] Wasternack C, Song S. Jasmonates: biosynthesis, metabolism, and signaling by proteins activating and repressing transcription. J Exp Bot. 2017;68:1303–21. 10.1093/jxb/erw443. 27940470 10.1093/jxb/erw443

[34] Chahar M, Kroupitski Y, Gollop R, Belausov E, Melotto M, Sela-Saldinger S. Determination of *Salmonella enterica* leaf internalization varies substantially according to the method and conditions used to assess bacterial localization. Front Microbiol. 2021;12:622068. 10.3389/fmicb.2021.622068. 34803936 10.3389/fmicb.2021.622068 PMC8603913

[35] Kroupitski Y, Golberg D, Belausov E, Pinto R, Swartzberg D, Granot D. Sela (Saldinger) S. Internalization of *Salmonella enterica* in leaves is induced by light and involves chemotaxis and penetration through open stomata. Appl Environ Microbiol. 2009;75:6076–86. 10.1128/AEM.01084-09. 19648358 10.1128/AEM.01084-09 PMC2753090

[36] Kroupitski Y, Gollop R, Belausov E, Pinto R, Sela (Saldinger) S, editors. *Salmonella enterica* growth conditions influence lettuce leaf internalization. Front Microbiol. 2019;10:639. 10.3389/fmicb.2019.00639 10.3389/fmicb.2019.00639 PMC6482241 31057491

[37] Jechalke S, Schierstaedt J, Becker M, Flemer B, Grosch R, Smalla K, Schikora A. *Salmonella* establishment in agricultural soil and colonization of crop plants depend on soil type and plant species. Front Microbiol. 2019;10:967. 10.3389/fmicb.2019.00967. 31156568 10.3389/fmicb.2019.00967 PMC6529577

[38] Roy D, Melotto M. Stomatal response and human pathogen persistence in leafy greens under preharvest and postharvest environmental conditions. Postharvest Biol Tech. 2019;148:76–82. 10.1016/j.postharvbio.2018.10.013.

[39] Saldaña Z, Sánchez E, Xicohtencatl-Cortes J, Puente JL, Girón JA. Surface structures involved in plant stomata and leaf colonization by Shiga-toxigenic *Escherichia coli* O157:H7. Front Microbiol. 2011;27:119. 10.3389/fmicb.2011.00119. 10.3389/fmicb.2011.00119 PMC3157101 21887151

[40] Leveau JHJ. A brief from the leaf: latest research to inform our Understanding of the phyllosphere Microbiome. Curr Opin Microbiol. 2019;49:41–9. 10.1016/j.mib.2019.10.002. 31707206 10.1016/j.mib.2019.10.002

[41] Thomas G, Kay WT, Fones HN. Life on a leaf: the epiphyte to pathogen continuum and interplay in the phyllosphere. BMC Biol. 2024;22:168. 10.1186/s12915-024-01967-1. 39113027 10.1186/s12915-024-01967-1 PMC11304629

[42] Cuggino SG, Posada-Izquierdo G, Bascón Villegas I, Theumer MG, Pérez-Rodríguez F. Effects of Chlorine and peroxyacetic acid wash treatments on growth kinetics of Salmonella in fresh-cut lettuce. Food Res Int. 2023;167:112451. 10.1016/j.foodres.2022.112451. 37087200 10.1016/j.foodres.2022.112451

[43] Ge C, Bohrerova Z, Lee J. Inactivation of internalized *Salmonella* Typhimurium in lettuce and green onion using ultraviolet C irradiation and chemical sanitizers. J Appl Microbiol. 2013;114:1415–24. 10.1111/jam.12154. 23351161 10.1111/jam.12154

[44] Niemira BA. Irradiation compared with chlorination for elimination of *Escherichia coli* O157:H7 internalized in lettuce leaves: influence of lettuce variety. J Food Sci. 2008;73:208–13. 10.1111/j.1750-3841.2008.00746.x. 10.1111/j.1750-3841.2008.00746.x 18577002

[45] Oblessuc PR, Matiolli CC, Melotto M. Novel molecular components involved in callose-mediated Arabidopsis defense against *Salmonella enterica* and *Escherichia coli* O157:H7. BMC Plant Biol. 2020;20:16. 10.1186/s12870-019-2232-x. 31914927 10.1186/s12870-019-2232-x PMC6950905

[46] Schikora A, Carreri A, Charpentier E, Hirt H. The dark side of the salad: *Salmonella typhimurium* overcomes the innate immune response of Arabidopsis thaliana and shows an endopathogenic lifestyle. PLoS ONE. 2008;3:e2279. 10.1371/journal.pone.0002279. 18509467 10.1371/journal.pone.0002279 PMC2386236

[47] Cowles KN, Iyer AS, McConnell I, Guillemette EG, Nellore D, Zaacks SC, Barak JD. Established *Pseudomonas syringae* Pv. *tomato* infection disrupts immigration of leaf surface bacteria to the Apoplast. Front Microbiol. 2025;16:1546411. 10.3389/fmicb.2025.1546411. 39963495 10.3389/fmicb.2025.1546411 PMC11830748

[48] Dixon MH, Cowles KN, Zaacks SC, Marciniak IN, Barak JD. *Xanthomonas* infection transforms the Apoplast into an accessible and habitable niche for *Salmonella enterica*. Appl Environ Microbiol. 2022;88:e0133022. 10.1128/aem.01330-22. 36314834 10.1128/aem.01330-22 PMC9680631

[49] Dixon MH, Nellore D, Zaacks SC, Barak JD. Time of arrival during plant disease progression and humidity additively influence *Salmonella enterica* colonization of lettuce. Appl Environ Microbiol. 2024;90:e01311–24. 10.1128/aem.01311-24. 39207142 10.1128/aem.01311-24 PMC11409676

[50] Student J, Weitz T, Blewett T, Yaron S, Melotto M. Lettuce genotype-dependent effects of temperature on *Escherichia coli* O157:H7 persistence and plant head growth. J Food Prot. 2024;87:100334. 10.1016/j.jfp.2024. 39074612 10.1016/j.jfp.2024.100334

[51] Johnson N, Litt PK, Kniel KE, Bais H. Evasion of plant innate defense response by *Salmonella* on lettuce. Front Microbiol. 2020;11:500. 10.3389/fmicb.2020.00500. 32318033 10.3389/fmicb.2020.00500 PMC7147383

[52] Qi J, Wang J, Gong Z, Zhou JM. Apoplastic ROS signaling in plant immunity. Curr Opin Plant Biol. 2017;38:92–100. 10.1016/j.pbi.2017.04.022. 28511115 10.1016/j.pbi.2017.04.022

[53] Lefevere H, Bauters L, Gheysen G. Salicylic acid biosynthesis in plants. Front Plant Sci. 2020;11:338. 10.3389/fpls.2020.00338. 32362901 10.3389/fpls.2020.00338 PMC7182001

[54] Torrens-Spence MP, Bobokalonova A, Carballo V, Glinkerman CM, Pluskal T, Shen A, Weng J-K. PBS3 and EPS1 complete Salicylic acid biosynthesis from isochorismate in Arabidopsis. Mol Plant. 2019;12:1577–86. 10.1016/j.molp.2019.11.005. 31760159 10.1016/j.molp.2019.11.005

[55] Liu Y, He C. Regulation of plant reactive oxygen species (ROS) in stress responses: learning from AtRBOHD. Plant Cell Rep. 2016;35:995–1007. 10.1007/s00299-016-1950-x. 26883222 10.1007/s00299-016-1950-x

[56] Ma M, Wang P, Chen R, Bai M, He Z, Xiao D, Xu G, Wu H, Zhou JM, Dou D, Bi G, Liang X. The OXIDATIVE SIGNAL-INDUCIBLE1 kinase regulates plant immunity by linking microbial pattern-induced reactive oxygen species burst to MAP kinase activation. Plant Cell. 2024;37:koae311. 10.1093/plcell/koae311. 39566103 10.1093/plcell/koae311 PMC11663599

[57] Brandl MT, Hua SST, Sarreal SBL. Association of *Escherichia coli* O157:H7 density change with hydrogen peroxide but not carbohydrate concentration in the leaf content of different lettuce types and spinach. Foods. 2025;14:709. 10.3390/foods14040709. 40002152 10.3390/foods14040709 PMC11854576

[58] German L, Yeshvekar R, Benitez-Alfonso Y. Callose metabolism and the regulation of cell walls and plasmodesmata during plant mutualistic and pathogenic interactions. Plant Cell Environ. 2022;46:391–404. 10.1111/pce.14510. 36478232 10.1111/pce.14510 PMC10107507

[59] Iniguez AL, Dong Y, Carter HD, Ahmer BM, Stone JM, Triplett EW. Regulation of enteric endophytic bacterial colonization by plant defenses. Mol Plant Microbe Interact. 2005;18:169–78. 10.1094/MPMI-18-0169. 15720086 10.1094/MPMI-18-0169

[60] Dong D, Yang Z, Ma Y, Li S, Wang M, Li Y, Liu Z, Jia C, Han L, Chao Y. Expression of a hydroxycinnamoyl-Coa Shikimate/quinate hydroxycinnamoyl transferase 4 gene from Zoysia japonica (ZjHCT4) causes excessive elongation and lignin composition changes in *Agrostis stolonifera*. Int J Mol Sci. 2022;23:9500. 10.3390/ijms23169500. 36012757 10.3390/ijms23169500 PMC9408870

[61] Serrani-Yarce JC, Escamilla-Trevino L, Barros J, Gallego-Giraldo L, Pu Y, Ragauskas A, Dixon RA. Targeting hydroxycinnamoyl CoA: Shikimate hydroxycinnamoyl transferase for lignin modification in *Brachypodium distachyon*. Biotechnol Biofuels. 2021;1450. 10.1186/s13068-021-01905-1. 10.1186/s13068-021-01905-1 PMC7913460 33640016

[62] Ali B. Salicylic acid: an efficient elicitor of secondary metabolite production in plants. Agric Biotechnol. 2021;31:101884. 10.1016/j.bcab.2020.101884.

[63] Marvasi M, Noel JT, George AS, Farias MA, Jenkins KT, Hochmuth G, Xu Y, Giovanonni JJ, Teplitski M. Ethylene signalling affects susceptibility of tomatoes to Salmonella. Microb Biotechnol. 2014;7:545–55. 10.1111/1751-7915.12130. 24888884 10.1111/1751-7915.12130 PMC4265073

[64] Kim MS, Cho SM, Kang EY, Im YJ, Hwangbo H, Kim YC, Ryu CM, Yang KY, Chung GC, Cho BH. Galactinol is a signaling component of the induced systemic resistance caused by *Pseudomonas chlororaphis* O6 root colonization. Mol Plant Microbe Interact. 2008;21:1643–53. 10.1094/MPMI-21-12-1643. 18986260 10.1094/MPMI-21-12-1643

[65] Meyer T, Vigouroux A, Aumont-Nicaise M, Comte G, Vial L, Lavire C, Moréra S. The plant defense signal galactinol is specifically used as a nutrient by the bacterial pathogen *Agrobacterium fabrum*. J Biol Chem. 2018;293:7930–41. 10.1074/jbc.RA118.001856. 29602905 10.1074/jbc.RA118.001856 PMC5971467

[66] Godson A, van der Hoorn RAL. The front line of defence: a meta-analysis of apoplastic proteases in plant immunity. J Exp Bot. 2021;72:3381–94. 10.1093/jxb/eraa602. 33462613 10.1093/jxb/eraa602 PMC8042752

[67] Oblessuc PR, Melotto M. A simple assay to assess *Salmonella enterica* persistence in lettuce leaves after low inoculation dose. Front Microbiol. 2020;11:1516. 10.3389/fmicb.2020.01516. 32765443 10.3389/fmicb.2020.01516 PMC7381196

[68] Schikora A, Virlogeux-Payant I, Bueso E, Garcia AV, Nilau T, Charrier A, Pelletier S, Menanteau P, Baccarini M, Velge P, Hirt H. Conservation of *Salmonella* infection mechanisms in plants and animals. PLoS ONE. 2011;6:e24112. 10.1371/journal.pone.0024112. 21915285 10.1371/journal.pone.0024112 PMC3167816

[69] Porwollik S, Santiviago CA, Cheng P, Long F, Desai P, Fredlund J, Srikumar S, Silva CA, Chu W, Chen X, Canals R, Reynolds MM, Bogomolnaya L, Shields C, Cui P, Guo J, Zheng Y, Endicott-Yazdani T, Yang HJ, Maple A, Ragoza Y, Blondel CJ, Valenzuela C, Andrews-Polymenis H, McClelland M. Defined single-gene and multi-gene deletion mutant collections in *Salmonella enterica* Sv typhimurium. PLoS ONE. 2014;9:e99820. 10.1371/journal.pone.0099820. 25007190 10.1371/journal.pone.0099820 PMC4089911

[70] Sperandio V, Torres AG, Girón JA, Kaper JB. Quorum sensing is a global regulatory mechanism in entrohemorrhagic *Escherichia coli* O157:H7. J Bac. 2001;183:5187–97. 10.1128/JB.183.17.5187-5197.2001. 10.1128/JB.183.17.5187-5197.2001 PMC95396 11489873

[71] Jacob C, Panchal S, Melotto M. Surface inoculation and quantification of *Pseudomonas syringae* population in the Arabidopsis leaf Apoplast. Bio Protoc. 2017;7:e2167. 10.21769/BioProtoc.2167. 28573169 10.21769/BioProtoc.2167 PMC5448416

[72] R Core Team. R: A language and environment for statistical computing. R Foundation for Statistical Computing, Vienna, Austria. 2020. https://www.R-project.org/

[73] Conway JR, Lex A, Gehlenborg N. UpSetR: an R package for the visualization of intersecting sets and their properties. Bioinformatics. 2017;33:2938–40. 10.1093/bioinformatics/btx364. 28645171 10.1093/bioinformatics/btx364 PMC5870712

[74] Alexa A, Rahnenführer J. Gene set enrichment analysis with topgo. Bioconductor Improv. 2009;27:1–26.

[75] Kanehisa M, Sato Y. KEGG mapper for inferring cellular functions from protein sequences. Protein Sci. 2020;29:28–35. 10.1002/pro.3711. 31423653 10.1002/pro.3711 PMC6933857

[76] Aramaki T, Blanc-Mathieu R, Endo H, Ohkubo K, Kanehisa M, Goto S, Ogata H. KofamKOALA: KEGG ortholog assignment based on profile HMM and adaptive score threshold. Bioinformatics. 2020;362251–2252. 10.1093/bioinformatics/btz859. 10.1093/bioinformatics/btz859 PMC7141845 31742321

[77] Chen L, Chu C, Lu J, Kong X, Huang T, Cai YD. Gene ontology and KEGG pathway enrichment analysis of a drug target-based classification system. PLoS ONE. 2015;10:e0126492. 10.1371/journal.pone.0126492. 25951454 10.1371/journal.pone.0126492 PMC4423955

[78] O’Leary BM, Rico A, McCraw S, Fones HN, Preston GM. The infiltration-centrifugation technique for extraction of apoplastic fluid from plant leaves using *Phaseolus vulgaris* as an example. J Vis Exp. 2014;94:e52113. 10.3791/52113. 10.3791/52113 PMC4396939 25549068

[79] Nouchi I, Hayashi K, Hiradate S, Ishikawa S, Fukuoka M, Chen CP, Kobayashi K. Overcoming the difficulties in collecting apoplastic fluid from rice leaves by the infiltration–centrifugation method. Plant Cell Physiol. 2012;53:1659–68. 10.1093/pcp/pcs102. 22813544 10.1093/pcp/pcs102

[80] Fiehn O. Metabolomics by gas chromatography– massspectrometry: combined targeted and untargeted profiling. Curr Protoc Mol Biol. 2016;114. 30.4.1–30.4.32. 10.1002/0471142727.mb3004s114 PMC4829120 27038389

[81] Fiehn O, Wohlgemuth G, Scholz M, Kind T, Lee DY, Lu Y, Moon S, Nikolau B. Quality control for plant metabolomics: reporting MSI-compliant studies. Plant J. 2008;53:691–704. 10.1111/j.1365-313X.2007.03387.x. 18269577 10.1111/j.1365-313X.2007.03387.x

[82] Fiehn O, Wohlgemuth G, Scholz M. Setup and annotation of metabolomics experiments by integrating biological and mass spectrometric metadata. Proc Lect Notes Bioinf. 2005;3615:224–39. 10.1007/11530084_18.

[83] Chong J, Wishart DS, Xia J. Using metaboanalyst 4.0 for comprehensive and integrative metabolomics data analysis. Curr Protoc Bioinf. 2019;68:e86. 10.1002/cpbi.86. 10.1002/cpbi.86 31756036

