# Supplementary material for: Lettuce immune responses and apoplastic metabolite profile contribute to reduced internal leaf colonization by human bacterial pathogens
Source: BMC Plant Biol. 2025 May 14;25:635. doi: 10.1186/s12870-025-06636-1 (PMC12076921; doi:10.1186/s12870-025-06636-1)
Supplement: Supplementary file 1 — Supplementary Material 1: Fig. S1. Photos of representative 4-week-old plants of the lettuce cultivars Green Towers (A), Lollo Rossa (B), and Red Tide (C) used in the experiments. [file 12870_2025_6636_MOESM1_ESM.pdf]

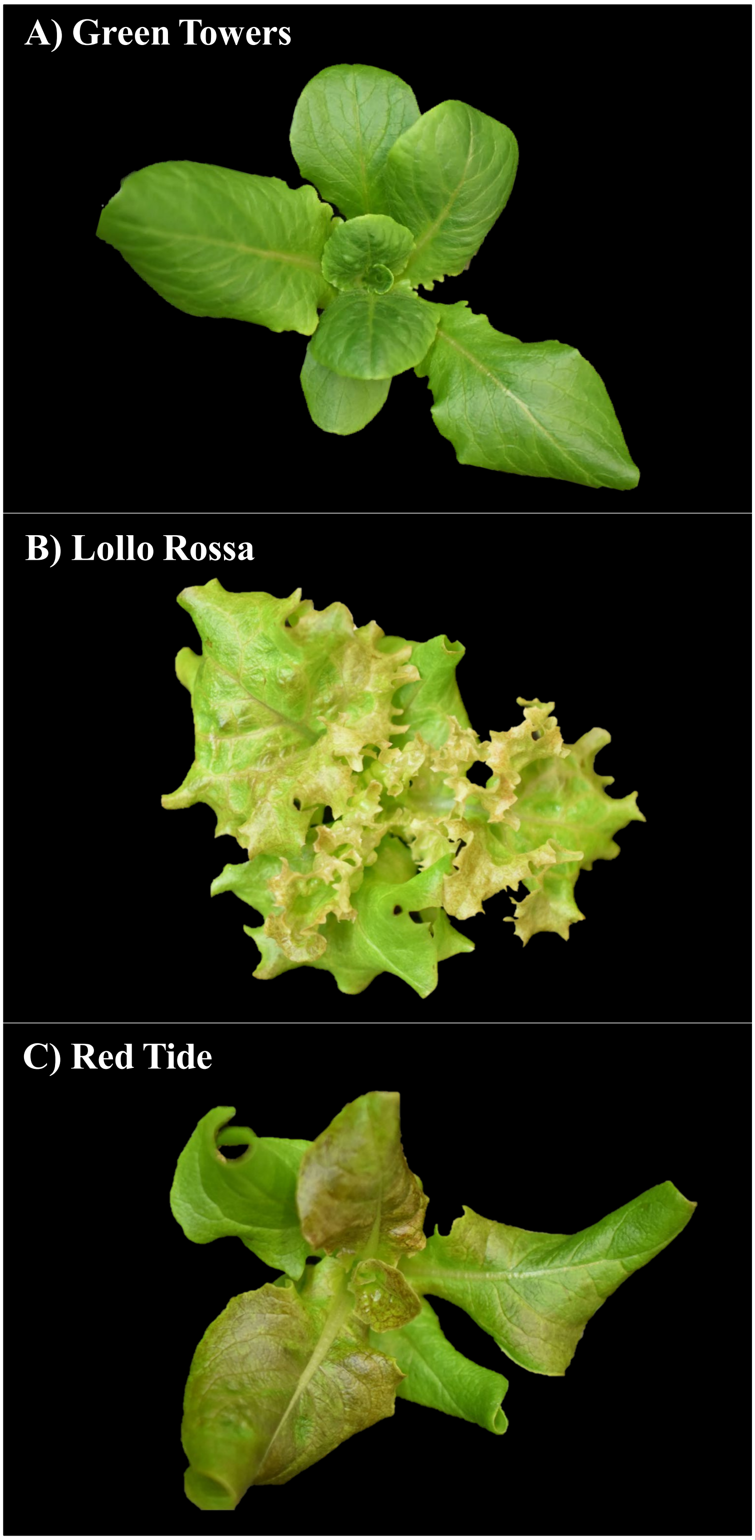

Fig. S1. Photos of representative 4-week-old plants of the lettuce cultivars Green Towers (**A**), Lollo Rossa (**B**), and Red Tide (**C**) used in the experiments.
