# Supplementary material for: Lettuce immune responses and apoplastic metabolite profile contribute to reduced internal leaf colonization by human bacterial pathogens
Source: BMC Plant Biol. 2025 May 14;25:635. doi: 10.1186/s12870-025-06636-1 (PMC12076921; doi:10.1186/s12870-025-06636-1)
Supplement: Supplementary file 4 — Supplementary Material 4: Fig. S2. Hierarchical clustering analysis of all detected transcripts based on Log2 fold change (FC) gene expression (bacterium vs. mock) for each lettuce cultivar and time point. Relative gene expression in Green Towers (GT), Lollo Rossa (LR), and Red Tide (RT) at 1- and 7-days post inoculation with Escherichia coli O157:H7 (Ec) or Salmonella enterica ser. Typhimurium 14028s (Se) is listed in Dataset S2. Heatmap and clustering were conducted using the pheatmap R package with default analysis settings. [file 12870_2025_6636_MOESM4_ESM.pdf]

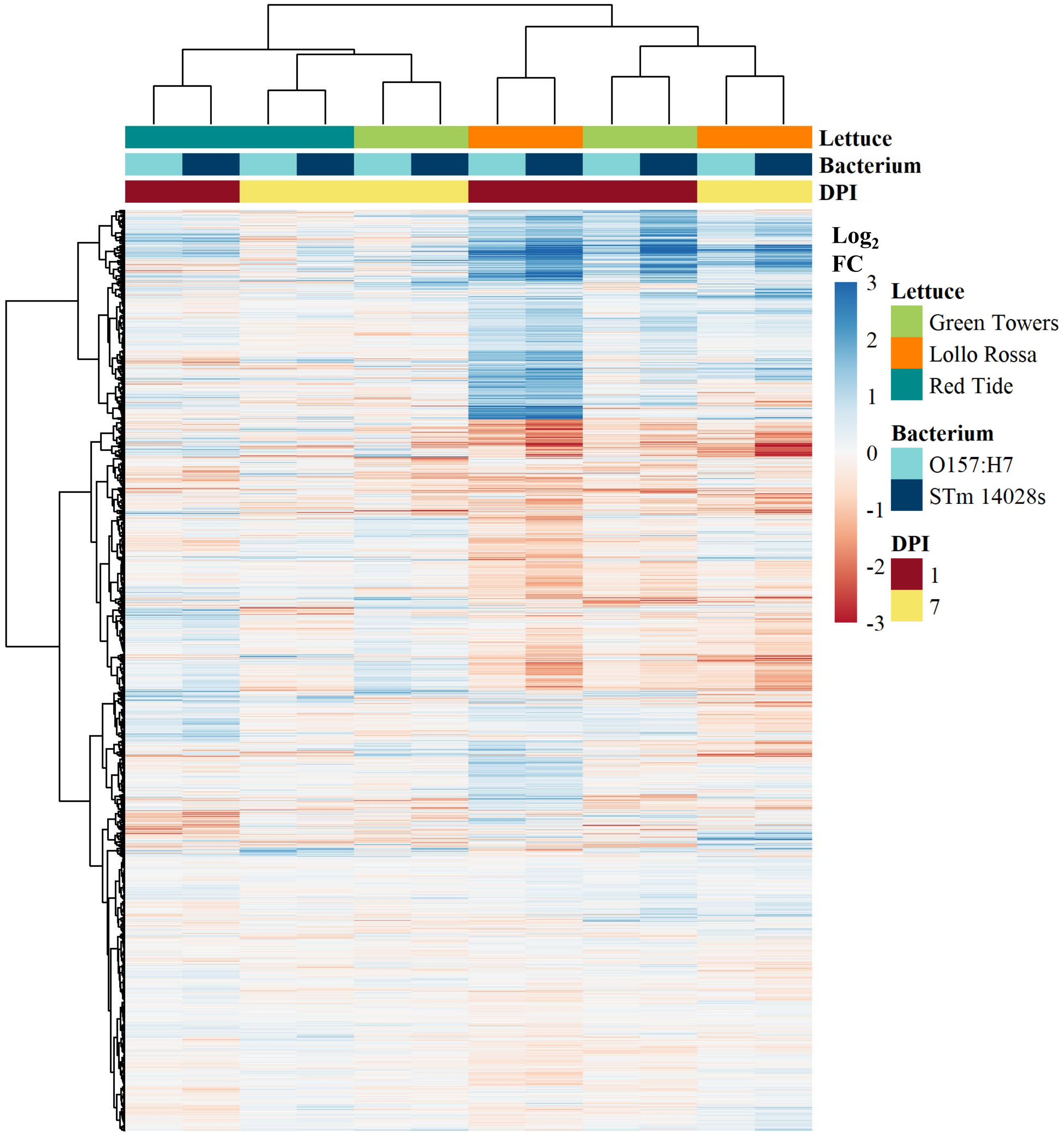

Fig. S2. Hierarchical clustering analysis of all detected transcripts based on Log_2 fold change (FC) gene expression (bacterium vs. mock) for each lettuce cultivar and time point. Relative gene expression in Green Towers (GT), Lollo Rossa (LR), and Red Tide (RT) at 1- and 7-days post inoculation with *Escherichia coli* O157:H7 (Ec) or *Salmonella enterica* ser. Typhimurium 14028s (Se) is listed in Dataset S2. Heatmap and clustering were conducted using the pheatmap R package with default analysis settings.
