# Supplementary material for: Lettuce immune responses and apoplastic metabolite profile contribute to reduced internal leaf colonization by human bacterial pathogens
Source: BMC Plant Biol. 2025 May 14;25:635. doi: 10.1186/s12870-025-06636-1 (PMC12076921; doi:10.1186/s12870-025-06636-1)
Supplement: Supplementary file 5 — Supplementary Material 5: Fig. S3. Intersection analysis of differentially expressed genes (DEGs) in the lettuce cultivars Green Towers (GT), Lollo Rossa (LR), and Red Tide (RT) at 1- and 7-days post inoculation (DPI) with Escherichia coli O157:H7 or Salmonella enterica ser. Typhimurium 14028s. Plots show the number of unique and common DEGs among the treatments for each cultivar (A-C) or for each bacterium (D, E) (X-axis) and eight (A-C) or ten (D, E) intersections exhibiting the highest number of DEGs (Y-axis) for both up and down regulated DEGs. Intersection analyses and plots were generated by using the UpSetR function in R. [file 12870_2025_6636_MOESM5_ESM.pdf]

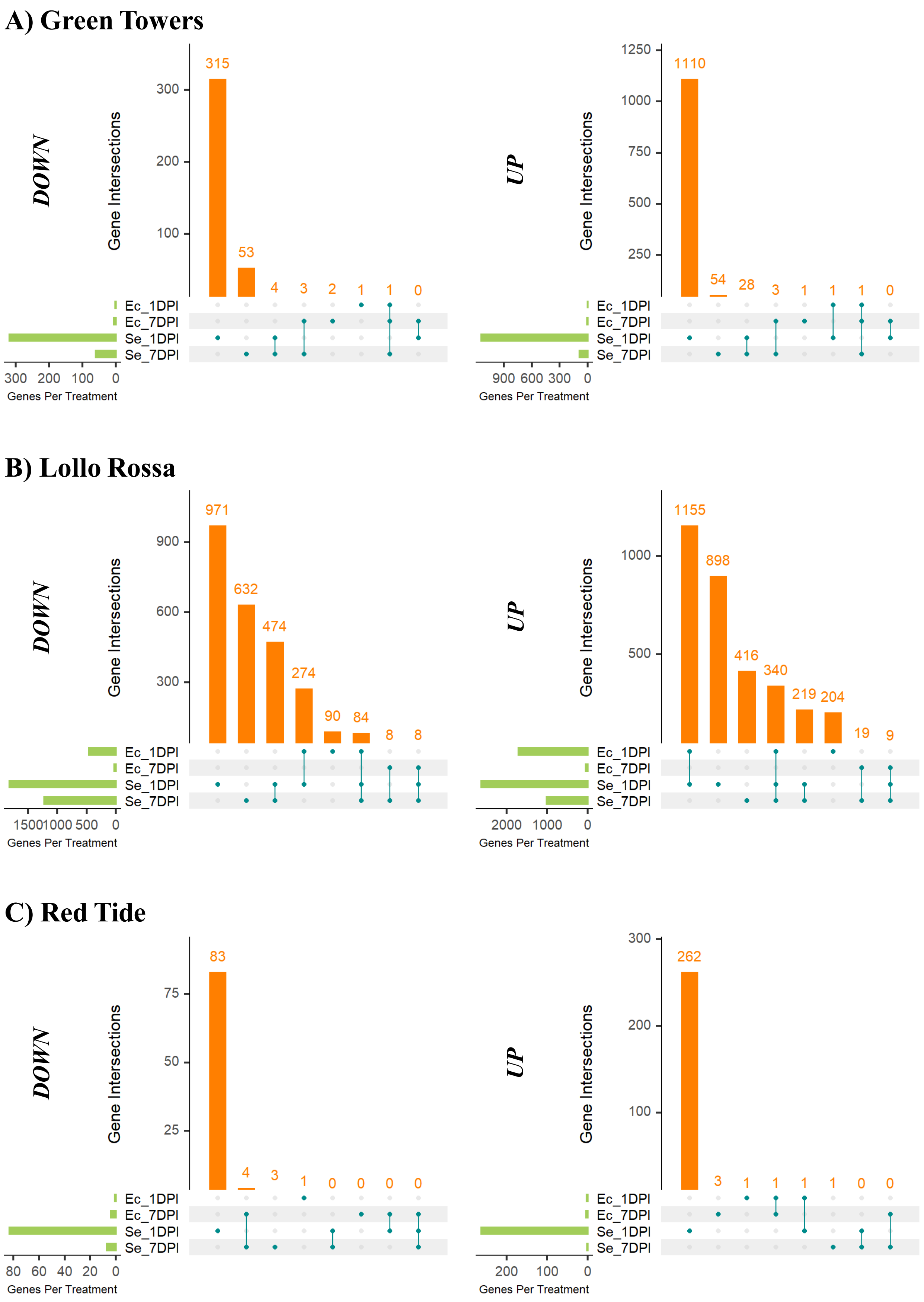
A) Green Towers
DOWN
Gene Intersections
315
53
4
3
2
1
1
0
Ec_1DPI
Ec_7DPI
Se_1DPI
Se_7DPI
300 200 100 0
Genes Per Treatment
UP
1110
54
28
3
1
1
1
0
900 600 300 0
B) Lollo Rossa
971
632
474
274
90
84
8
8
1500 1000 500 0
1155
898
416
340
219
204
19
9
2000 1000 0
C) Red Tide
83
4
3
1
0
0
0
0
80 60 40 20 0
262
3
1
1
1
1
0
0
200 100 0

## D) O157:H7

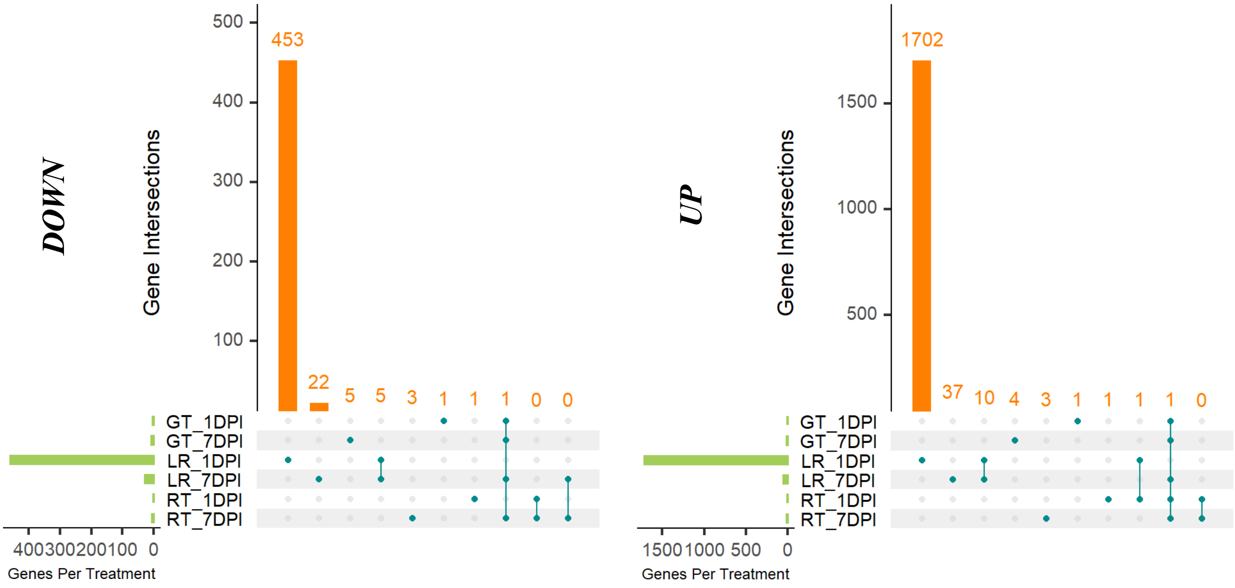


## E) STm 14028s

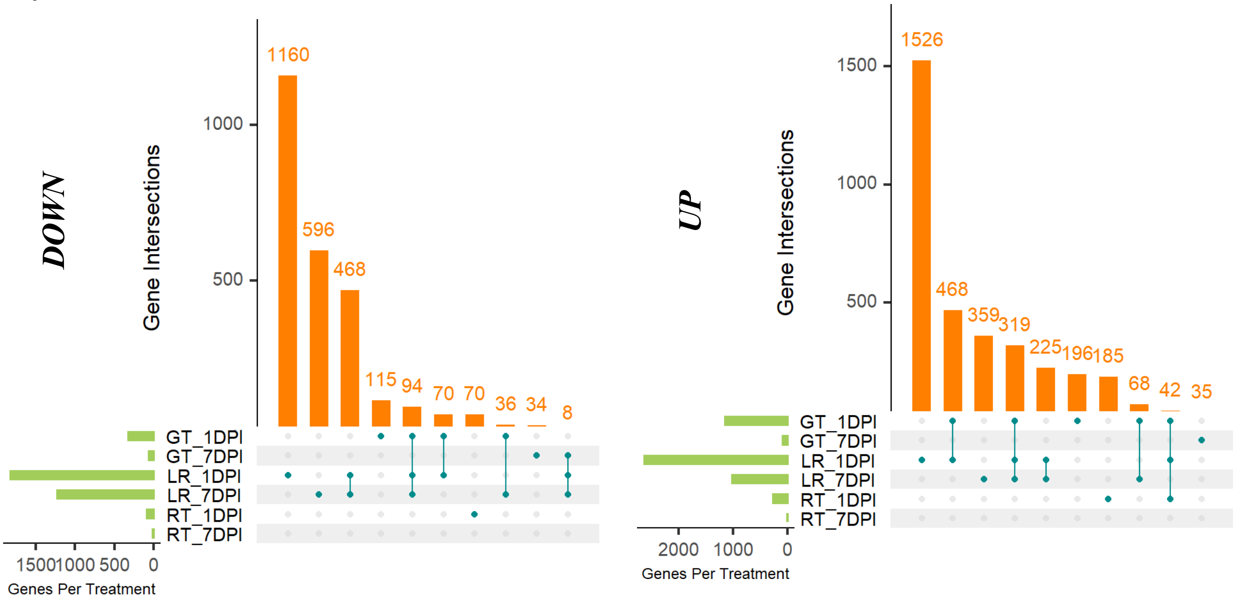


**Fig. S3.** Intersection analysis of differentially expressed genes (DEGs) in the lettuce cultivars Green Towers (GT), Lollo Rossa (LR), and Red Tide (RT) at 1- and 7-days post inoculation (DPI) with *Escherichia coli* O157:H7 or *Salmonella enterica* ser. Typhimurium 14028s. Plots show the number of unique and common DEGs among the treatments for each cultivar (A-C) or for each bacterium (D, E) (X-axis) and eight (A-C) or ten (D, E) intersections exhibiting the highest number of DEGs (Y-axis) for both up and down regulated DEGs. Intersection analyses and plots were generated by using the UpSetR function in R.
