# Supplementary material for: Lettuce immune responses and apoplastic metabolite profile contribute to reduced internal leaf colonization by human bacterial pathogens
Source: BMC Plant Biol. 2025 May 14;25:635. doi: 10.1186/s12870-025-06636-1 (PMC12076921; doi:10.1186/s12870-025-06636-1)
Supplement: Supplementary file 8 — Supplementary Material 8: Fig. S4. Reconstruction of lettuce KEGG metabolic pathways. To map the lettuce genes to KEGG pathways, the protein sequences of the corresponding annotated genome were used to obtain the KEGG protein IDs using the KofamKOALA BLAST tool (https://www.genome.jp/tools/kofamkoala/). Protein sequences with an E-value < 1x10^−5^ (Dataset S1) were used for the KEGG Mapper Reconstruct Pathway tool (https://www.kegg.jp/kegg/tool/map_pathway.html). All KEGG IDs were used to create lettuce reference metabolic routes (A), while the KEGG IDs of significantly differentially expressed genes (adjusted p-value < 0.05 and a Log2 fold change ≤ −1 or ≥ 1) were used to identify metabolic pathways modulated in the lettuce cultivars Green Towers (GT) and Lollo Rossa (LR) at 1- and 7-days post inoculation (DPI) with Salmonella enterica ser. Typhimurium 14028s or Escherichia coli O157:H7 (B). [file 12870_2025_6636_MOESM8_ESM.pdf]

A) Lettuce reference metabolism based on KEGG pathways

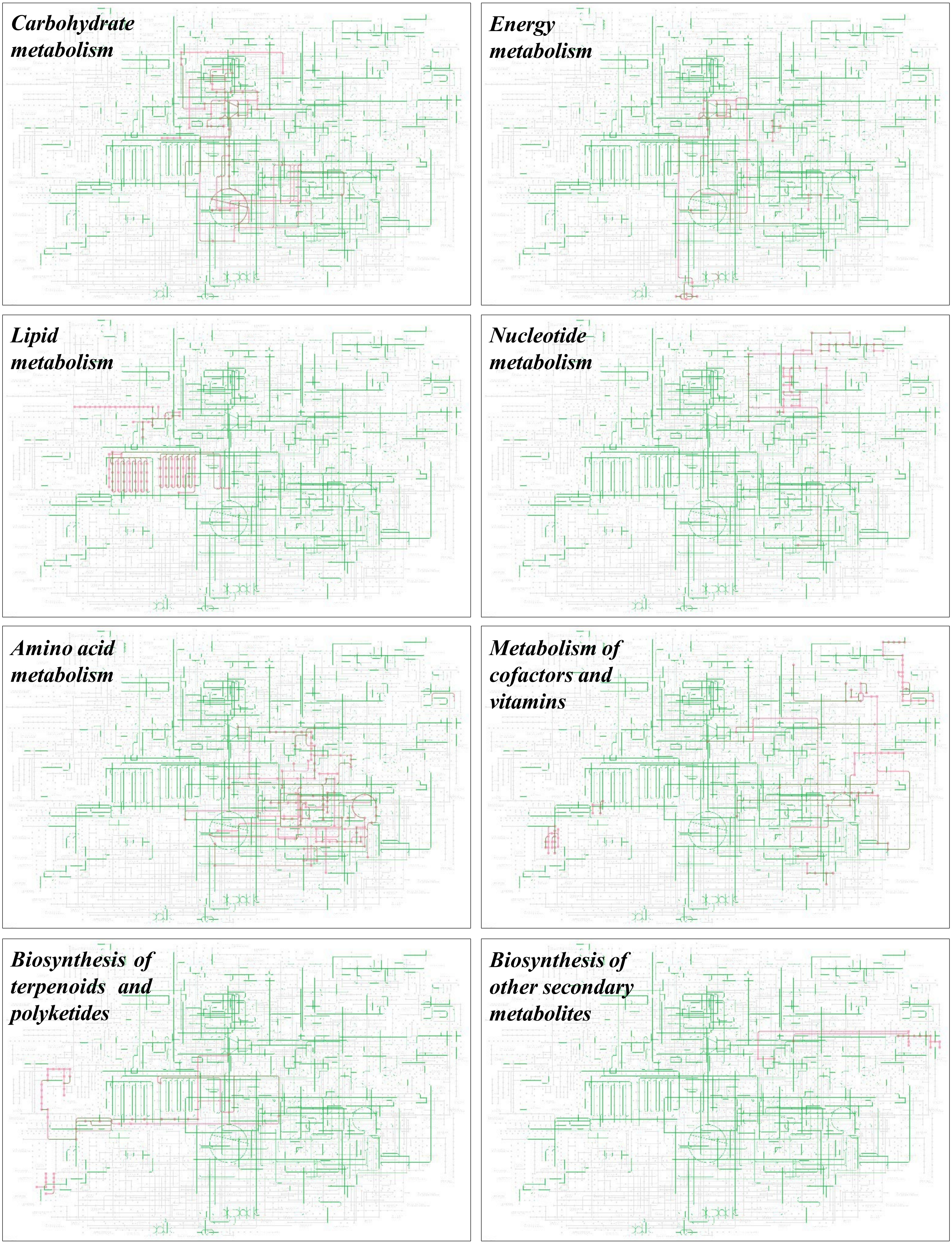

B) Lettuce metabolism modulated in each cultivar by each bacterium at 1 and 7 DPI

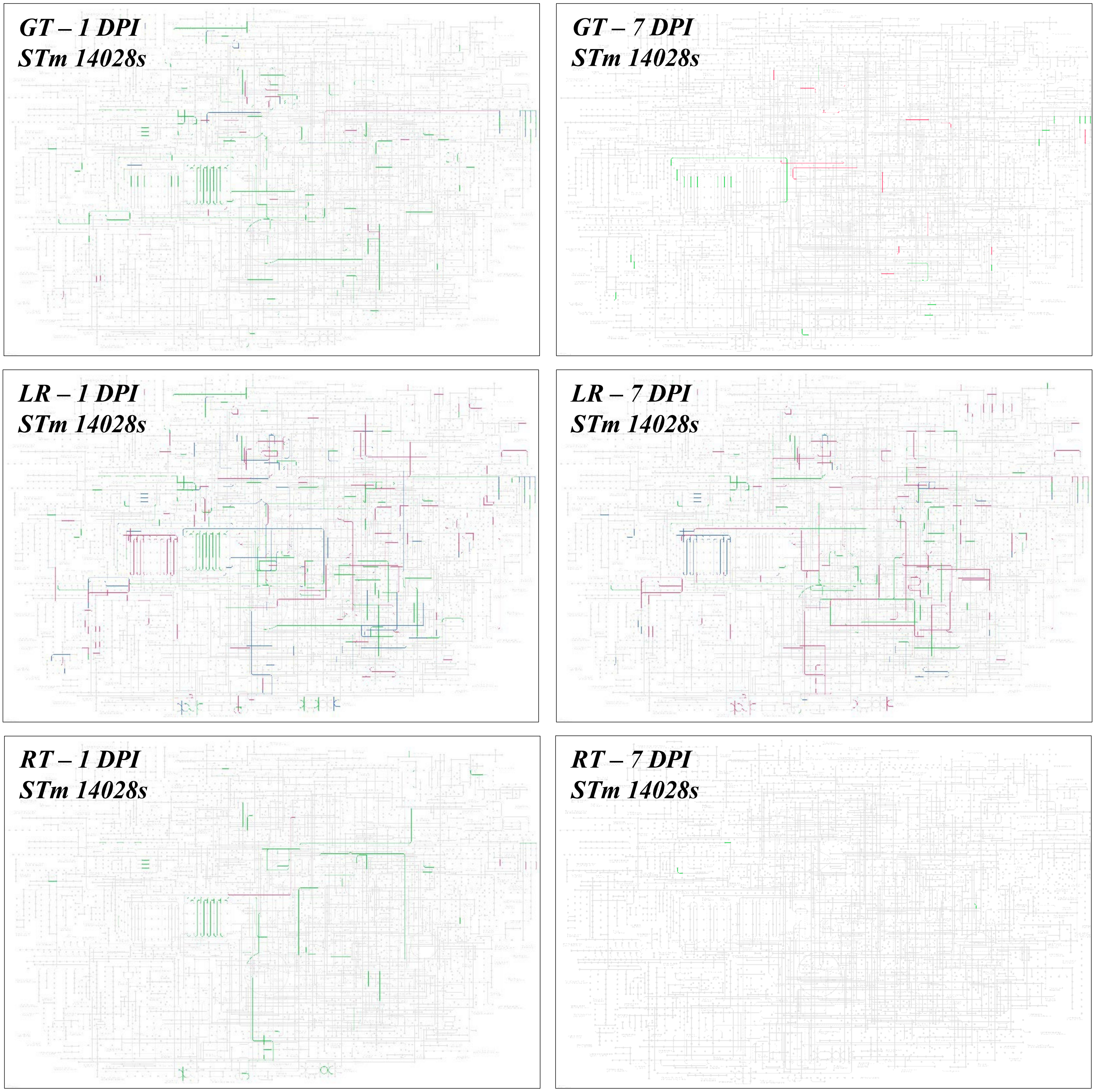

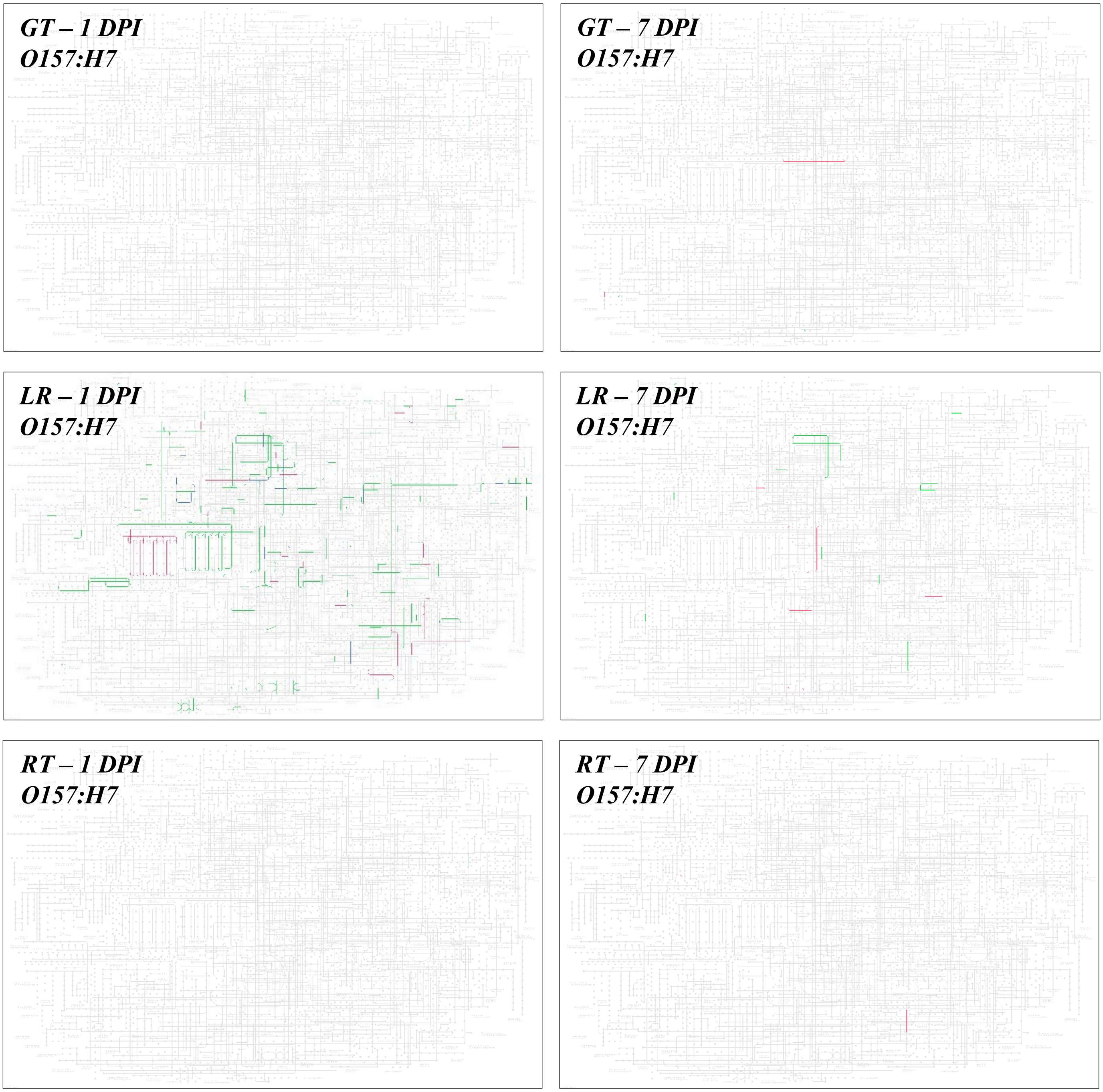

Fig. S4. Reconstruction of lettuce KEGG metabolic pathways. To map the lettuce genes to KEGG pathways, the protein sequences of the corresponding annotated genome were used to obtain the KEGG protein IDs using the KofamKOALA BLAST tool (https://www.genome.jp/tools/kofamkoala/). Protein sequences with an E-value < $1x10^{-5}$ (Dataset S1) were used for the KEGG Mapper Reconstruct Pathway tool (https://www.kegg.jp/kegg/tool/map_pathway.html). All KEGG IDs were used to create lettuce reference metabolic routes (**A**), while the KEGG IDs of significantly differentially expressed genes (adjusted p-value < 0.05 and a Log_2 fold change ≤ −1 or ≥ 1) were used to identify metabolic pathways modulated in the lettuce cultivars Green Towers (GT) and Lollo Rossa (LR) at 1- and 7-days post inoculation (DPI) with *Salmonella enterica* ser. Typhimurium 14028s or *Escherichia coli* O157:H7 (**B**).
