# Supplementary material for: Lettuce immune responses and apoplastic metabolite profile contribute to reduced internal leaf colonization by human bacterial pathogens
Source: BMC Plant Biol. 2025 May 14;25:635. doi: 10.1186/s12870-025-06636-1 (PMC12076921; doi:10.1186/s12870-025-06636-1)
Supplement: Supplementary file 10 — Supplementary Material 10: Fig. S5. Hierarchical clustering analysis of all 332 metabolites detected in the apoplastic wash fluid (AWF) collected from the lettuce cultivars Green Towers, Lollo Rossa, and Red Tide at 1- and 7-days post inoculation with Mock (Mk), Escherichia coli O157:H7 or Salmonella enterica ser. Typhimurium 14028s. Raw peak heights were normalized with the Log10 transformation and auto-scaling functions of MetaboAnalyst5.0 software. Values are listed in Dataset S5. Heatmaps and clustering were created with the pheatmap R package using default settings. [file 12870_2025_6636_MOESM10_ESM.pdf]

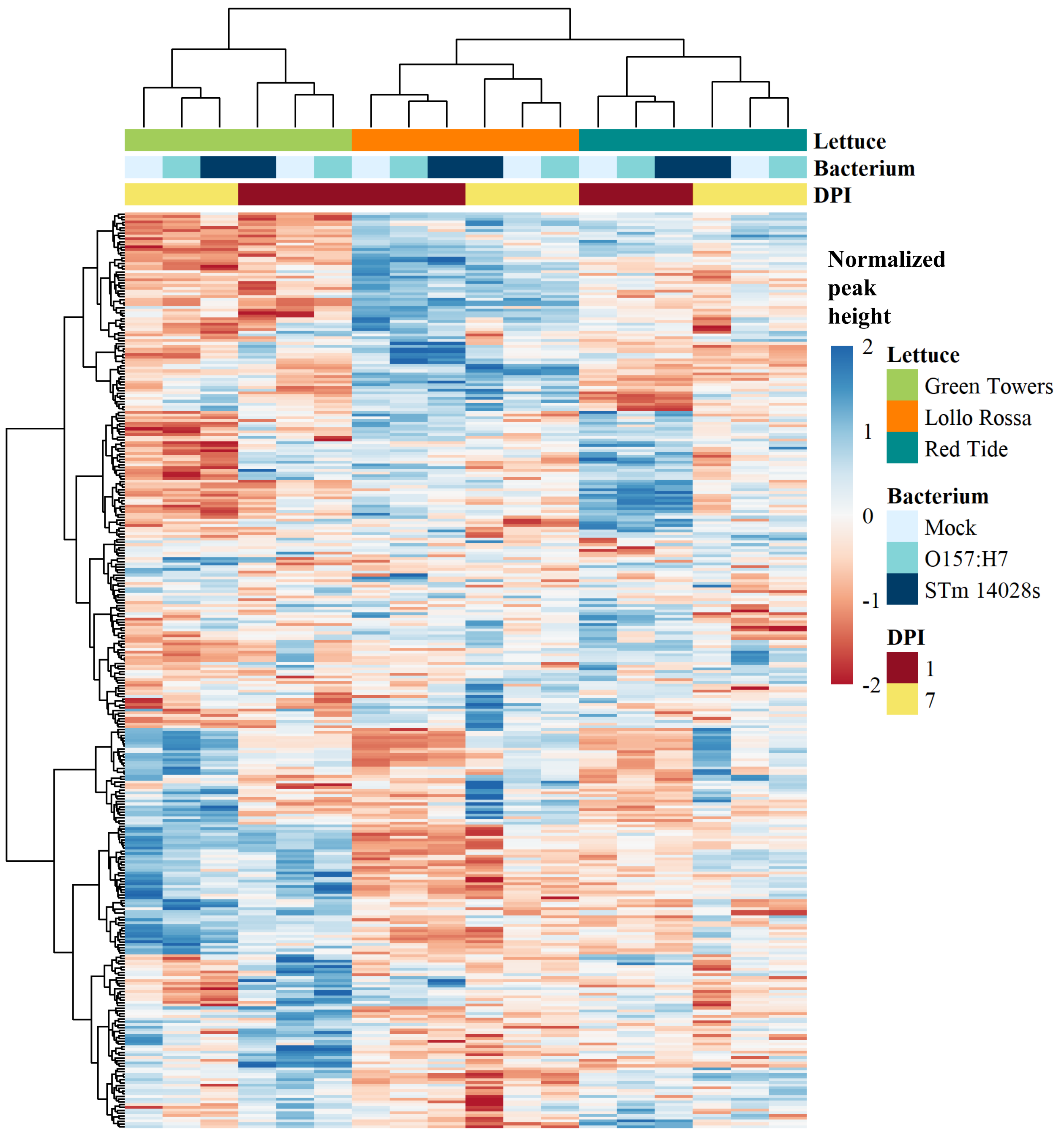

Fig. S5. Hierarchical clustering analysis of all 332 metabolites detected in the apoplastic wash fluid (AWF) collected from the lettuce cultivars Green Towers, Lollo Rossa, and Red Tide at 1- and 7-days post inoculation with *Escherichia coli* O157:H7 or *Salmonella enterica* ser. Typhimurium 14028s. Raw peak heights were normalized with the Log_{10} transformation and auto-scaling functions of MetaboAnalyst5.0 software. Values are listed in Dataset S5. Heatmaps and clustering were created with the pheatmap R package using default settings.
