# Supplementary material for: Lettuce immune responses and apoplastic metabolite profile contribute to reduced internal leaf colonization by human bacterial pathogens
Source: BMC Plant Biol. 2025 May 14;25:635. doi: 10.1186/s12870-025-06636-1 (PMC12076921; doi:10.1186/s12870-025-06636-1)
Supplement: Supplementary file 12 — Supplementary Material 12: Fig. S6. Multidimensional scaling (MDS) (A) and principal component analysis (PCA) (B) plots representing the correlation among biological replicates used for the whole leaf RNA-sequencing and AWF metabolomic analyses, respectively. Leaves of the lettuce cultivars Green Towers, Lollo Rossa, and Red Tide were vacuum infiltrated with mock (sterile distilled water) or bacterial inoculum containing 5 x 10^5^ CFU/mL of Escherichia coli O157:H7 or Salmonella enterica ser. Typhimurium 14028s. Leaves were sampled at 1- and 7-days post inoculation (DPI). Coordinates for MDS graphs were calculated using normalized read counts in the cmdscale function of the R software and the coordinates for PCA graphs were obtained by using normalized peak heights in the MetaboAnalyst 5.0 software. [file 12870_2025_6636_MOESM12_ESM.pdf]

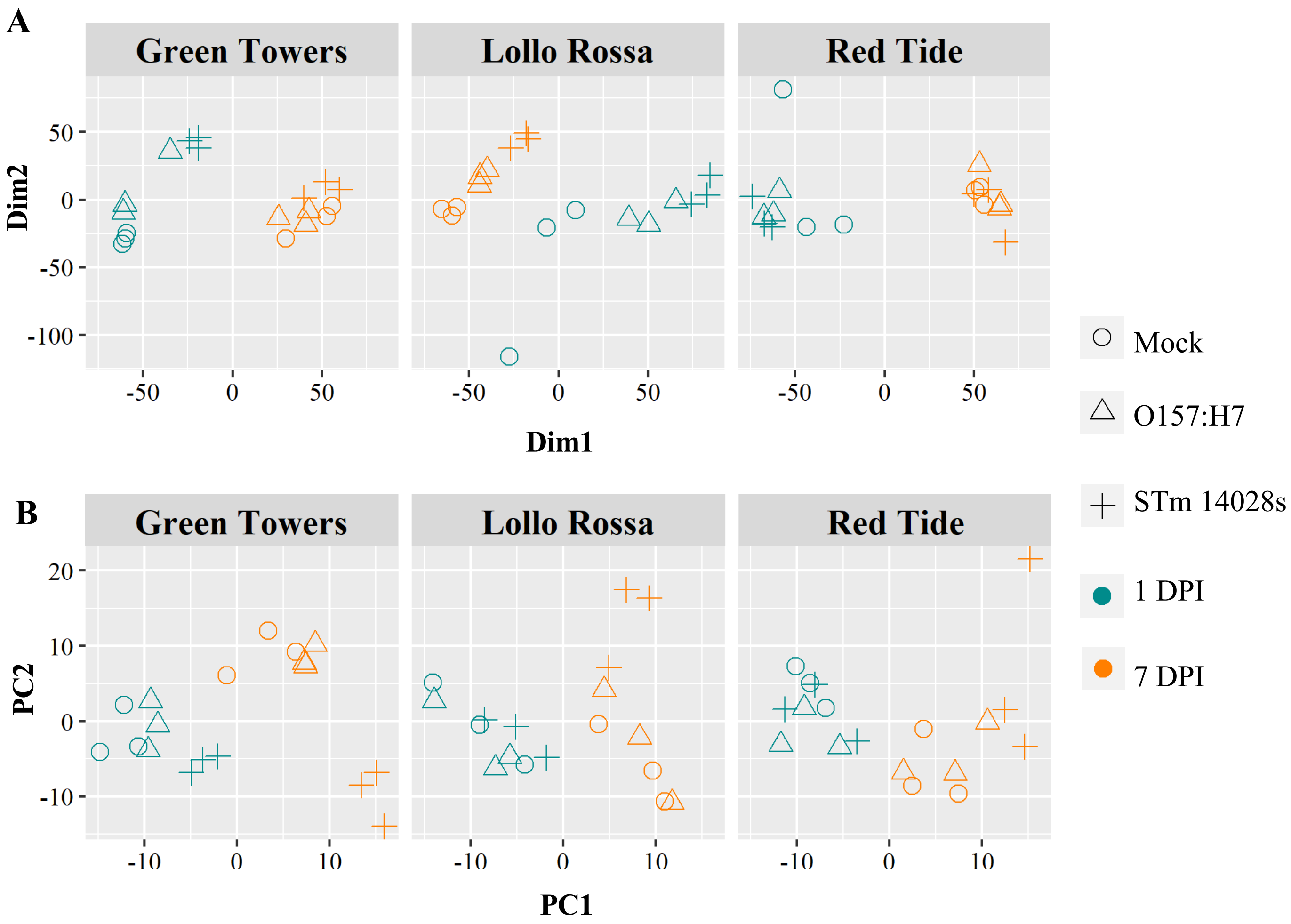

Fig. S6. Multidimensional scaling (MDS) **(A)** and principal component analysis (PCA) (**B**) plots representing the correlation among biological replicates used for the whole leaf RNA-sequencing and AWF metabolomic analyses, respectively. Leaves of the lettuce cultivars Green Towers, Lollo Rossa, and Red Tide were vacuum infiltrated with mock (sterile distilled water) or bacterial inoculum containing 5 x 10^5 CFU/mL of *Escherichia coli* O157:H7 or *Salmonella enterica* ser. Typhimurium 14028s. Leaves were sampled at 1- and 7-days post inoculation (DPI). Coordinates for MDS graphs were calculated using normalized read counts in the cmdscale function of the R software and the coordinates for PCA graphs were obtained by using normalized peak heights in the MetaboAnalyst 5.0 software.
