# Supplementary material for: Lettuce immune responses and apoplastic metabolite profile contribute to reduced internal leaf colonization by human bacterial pathogens
Source: BMC Plant Biol. 2025 May 14;25:635. doi: 10.1186/s12870-025-06636-1 (PMC12076921; doi:10.1186/s12870-025-06636-1)
Supplement: Supplementary file 13 — Supplementary Material 13: Fig. S7. Optimization of the apoplastic wash fluid (AWF) extraction procedure for each lettuce cultivar, Lollo Rossa, Red Tide, or Green Towers. (A) Graph shows the volume of infiltrated water (IW) and extracted AWF by gram of leaf tissue. The infiltrated volume of water was calculated by subtracting the initial leaf weight to the weight after infiltration. Pairwise mean comparison (IW versus AWF, for each centrifugation force) was performed with two-tail Student’s t-test (ns = not significant; * = p < 0.001; ** = p < 0.0001). (B) Graph shows the electric conductivity (EC) of the AWF at different centrifugation forces (xg). The effect of different centrifugation forces on the EC of the AWF was assessed through ANOVA followed by Tukey’s test (different letters on top of the boxes indicate statistically differences among the means). For both graphs, a replicate consisted of AWF collected and pooled from four leaves of two plants, and 8 plants were used for each sampling point (n = 4). [file 12870_2025_6636_MOESM13_ESM.pdf]

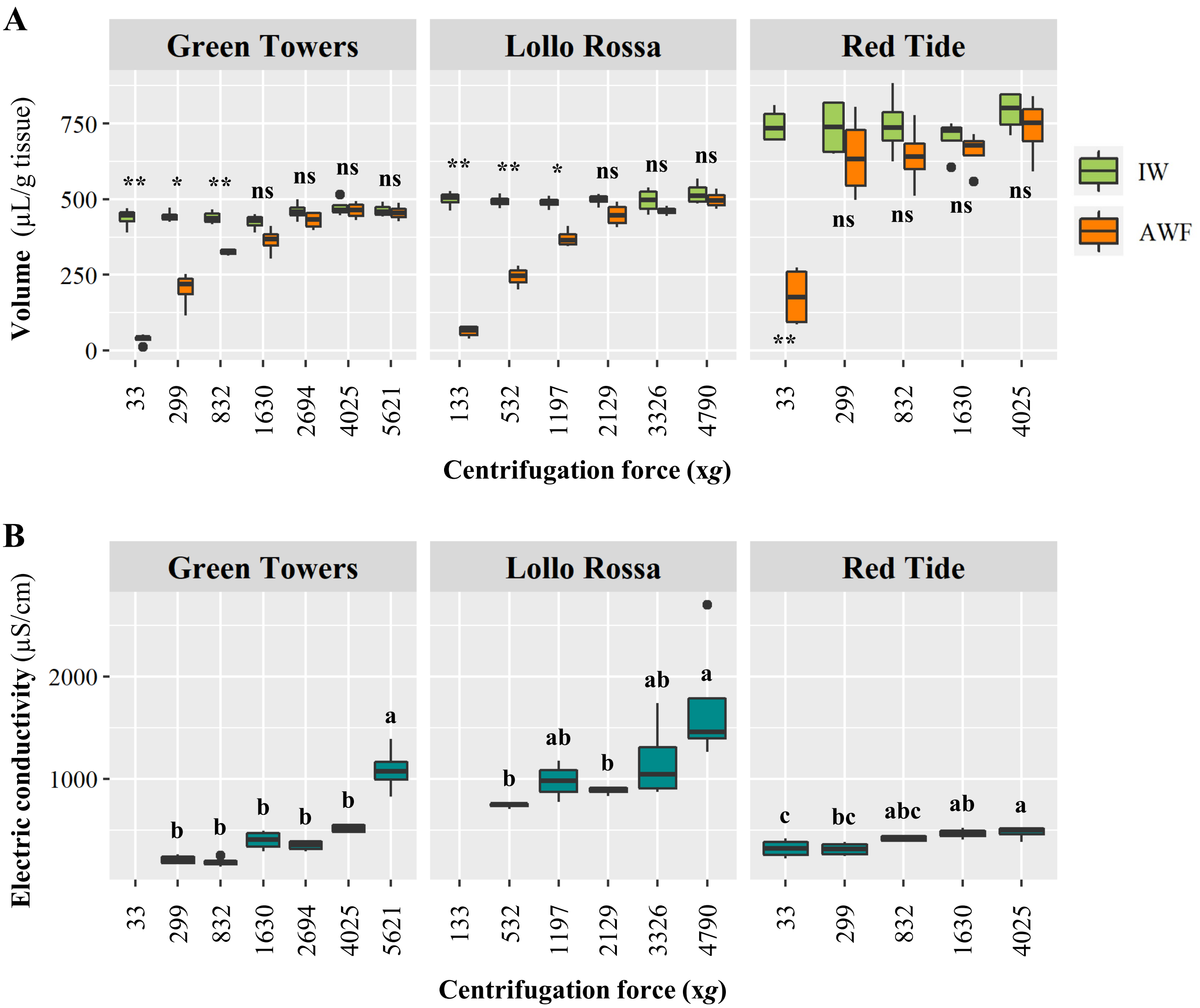

Fig. S7. Optimization of the apoplastic wash fluid (AWF) extraction procedure for each lettuce cultivar, Lollo Rossa, Red Tide, or Green Towers. **(A)** Graph shows the volume of infiltrated water (IW) and extracted AWF by gram of leaf tissue. The infiltrated volume of water was calculated by subtracting the initial leaf weight to the weight after infiltration. Pairwise mean comparison (IW versus AWF, for each centrifugation force) was performed with two-tail Student's *t*-test (ns = not significant; * = $p < 0.001$; ** = $p < 0.0001$). (**B**) Graph shows the electric conductivity (EC) of the AWF at different centrifugation forces (x*g*). The effect of different centrifugation forces on the EC of the AWF was assessed through ANOVA followed by Tukey's test (different letters on top of the boxes indicate statistically differences among the means). For both graphs, a replicate consisted of AWF collected and pooled from four leaves of two plants, and 8 plants were used for each sampling point (n = 4).
