# Supplementary material for: Lettuce immune responses and apoplastic metabolite profile contribute to reduced internal leaf colonization by human bacterial pathogens
Source: BMC Plant Biol. 2025 May 14;25:635. doi: 10.1186/s12870-025-06636-1 (PMC12076921; doi:10.1186/s12870-025-06636-1)
Supplement: Supplementary file 14 — Supplementary Material 14: Fig. S8. Evaluation of cytoplasmatic contamination of apoplastic wash fluid (AWF) samples based on regression analysis relative to the NADH standard curve. Temporal accumulation (X-axis) of NADH due to the enzymatic activity of cytoplasmic glucose-6-phosphate dehydrogenase in AWF recovered from non-inoculated leaves of the indicated lettuce cultivar. Note that at the end of the assay (20 min) the NADH accumulation in the AFW samples was comparable to the negative control provided with the kit. The plot shows data from two independent experiments (n = 6). [file 12870_2025_6636_MOESM14_ESM.pdf]

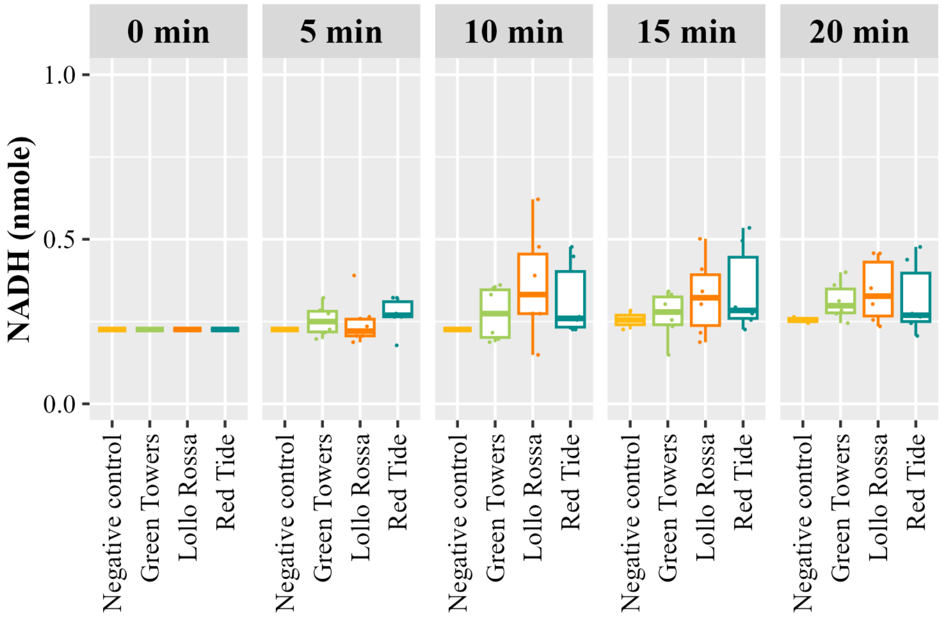

Fig. S8. Evaluation of cytoplasmatic contamination of apoplastic wash fluid (AWF) samples based on regression analysis relative to the NADH standard curve. Temporal accumulation (X-axis) of NADH due to the enzymatic activity of cytoplasmic glucose-6-phosphate dehydrogenase in AWF recovered from non-inoculated leaves of the indicated lettuce cultivar. Note that at the end of the assay (20 min) the NADH accumulation in the AFW samples was comparable to the negative control provided with the kit. Plot shows data from two independent experiments (n = 6).
